# Highly Efficient UV-Activated TiO_2_/SnO_2_ Surface Nano-matrix Gas Sensor: Enhancing Stability for ppb-Level NO_*x*_ Detection at Room Temperature

**DOI:** 10.1021/acsami.4c19998

**Published:** 2025-02-19

**Authors:** Moumita Deb, Youssef Ghossoub, Laurent Noel, Pin-Hsuan Li, Hsu-Yang Tsai, Olivier Soppera, Hsiao-Wen Zan

**Affiliations:** † International Ph.D. Program in Photonics, College of Electrical and Computer Engineering, National Yang Ming Chiao Tung University, 1001 Ta Hsueh Rd., Hsinchu 300093, Taiwan; ‡ Department of Photonics, National Yang Ming Chiao Tung University, 1001 Ta Hsueh Rd., Hsinchu 300093, Taiwan; § Department of Photonics, National Chiao Tung University, 1001 Ta Hsueh Rd., Hsinchu 300093, Taiwan; ∥ Université de Haute-Alsace, CNRS, IS2M UMR 7361, F-68100 Mulhouse, France; ⊥ Université de Strasbourg, F-67000 Strasbourg, France

**Keywords:** nanoporous TiO_2_/SnO_2_, photocatalyst, NO_*x*_, ppb, UV illumination, stability, room temperature

## Abstract

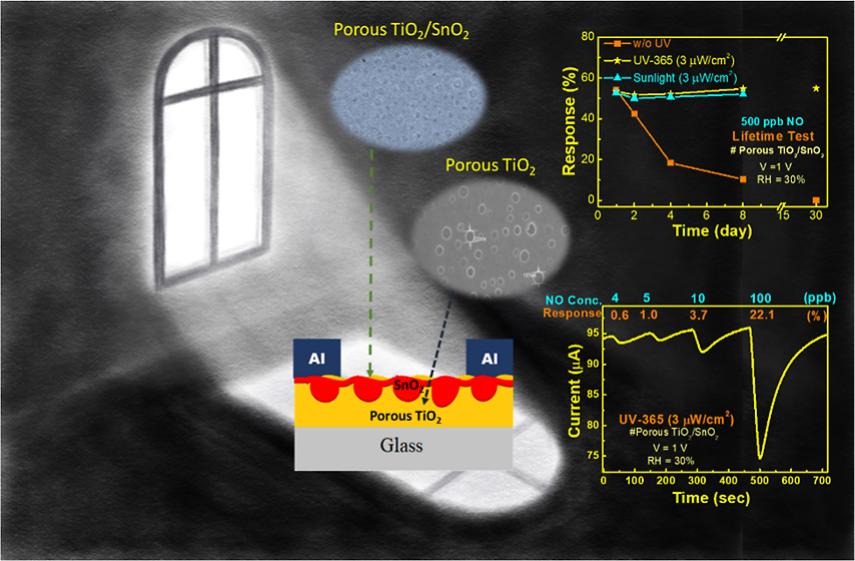

This study presents a new nanoporous TiO_2_/SnO_2_ heterojunction for NO_*x*_ gas detection by using a two-step sol–gel process. The unique TiO_2_ and SnO_2_ nanoheterojunction matrix right on the film surface enables the TiO_2_ photocatalyst to absorb minimal UV power (3 μW/cm^2^) and effectively transfer electrons to the SnO_2_ conduction band. The sensor detects NO and NO_2_ gases down to 4 ppb (response of 0.6%) and 10 ppb (response of 1.3%) at 1 V at room temperature. It also exhibits a fast recovery time (100 ± 40 s at 500 ppb NO_*x*_), an improved response over a wide relative humidity range (10–60%), and a long lifetime over 30 days. The ultralow UV power required can be easily harvested from sunlight, eliminating the need for UV LEDs. XPS and SEM analyses indicated that the unique nanoporous TiO_2_/SnO_2_ structure improves sensing performance, with oxygen vacancies playing a critical role in the NO_*x*_ gas sensing mechanism. This work demonstrated the highly efficient UV catalyst effect in sensors with the surface heterojunction matrix. The low-power ppb-level NO_*x*_ detection is suitable for environmental monitoring and respiratory disease detection.

## Introduction

In recent years, harmful pollutant gases such as NO_*x*_, CO, H_2_S, SO_2_, and NH_3_ have significantly increased in the environment due to globalization. Among these, NO_*x*_ (NO and NO_2_)^1^ stands out as a major pollutant emitted from industrial combustion processes and the burning of fossil fuels. It contributes to acid rain, photochemical smog, the greenhouse effect, PM2.5 (particulate matter), and ozone layer depletion.^2^ Exposure to NO_*x*_ gas can lead to various health problems including respiratory diseases, lung irritation, cardiovascular diseases, and cancer. The detection of NO_*x*_ gas at the parts per billion (ppb) level serves as an important biomarker for identifying lung diseases^3^ such as bronchitis and asthma.^4^ According to the U.S. Environmental Protection Agency (EPA), the health-based National Ambient Air Quality Standard (NAAQS) for NO_2_ is a maximum concentration of 100 ppb for 1 h daily, with an annual standard set at 53 ppb.^5^ Thus, environmental regulations worldwide necessitate monitoring and controlling NO_2_ emissions to comply with air quality standards, requiring the detection of ppb-level NO_2_ gas. Moreover, fractional exhaled NO levels in healthy patients below 25 ppb are considered normal, while for asthma patients, levels between 25 and 50 ppb are classified as an intermediate stage, and levels surpassing 50 ppb are deemed high.^6^ Detecting NO gas at the ppb level is crucial for the timely prevention of dangerous diseases. Therefore, selective detection of both NO and NO_2_ gases (NO_*x*_) at the parts-per-billion level is highly essential for monitoring environmental pollution and ensuring human safety.

Numerous methods exist for detecting NO_*x*_ gas, with common techniques including absorption spectroscopy,^7^ colorimetric,^8^ chemiluminescence,^9^ electrochemical,^10^ and chemoresistive/conductometric^11,12^ methods. Among these, the chemoresistive/conductometric technique stands out for its cost-effectiveness, simple fabrication process, and high reproducibility. Although there are several gas sensors that can operate at room temperature, such as MXene-based^13^ NO_2_ gas sensors with a limit of detection (LOD) of 11.0 ppb (experimentally 0.1 ppm) and SnSe-based^14^ NO_2_ sensors with a LOD of 345 ppb (experimentally 500 ppb), the metal oxide semiconductors (MOx)-based sensors show remarkable achievements due to their high sensitivity, excellent stability, and scalability. MOx are favored for gas detection applications because of their cost-effectiveness, tunable electronic properties, and ability to detect a wide range of gases, even at low concentrations. MOx-based sensors, such as those employing SnO_2_, ZnO, and WO_3_, demonstrate the capacity for detecting NO_*x*_ gas at ppb levels.^15^ However, these sensors typically operate at high temperatures, resulting in increased power consumption and limited portability. Note that recent advancements in wearable, flexible, and stretchable NO_2_ sensors also shift the focus toward developing room-temperature sensors with low-power-consumption technologies.^13,16,17^

To address these challenges, researchers are exploring UV illumination to enhance sensor performance at RT. UV illumination, by generating electron–hole pairs, increases oxygen adsorption on the sensing material, enhancing gas-sensing reactions and improving sensitivity, response time, and stability at RT (Table S1).^18−23^ For example, TiO_2_@SnO_2_ sensors prepared via hydrothermal methods^18^ or atomic layer deposition (ALD)^19^ detect HCHO gas at limits of detection (LOD) of 100 ppb^18^ and 1 ppm^19^, respectively, under UV irradiation. Similarly, TiO_2_@NGQDs^20^ detect NO at 10 ppm. AuNP-decorated ZnO/TiO_2_ nanorods^21^ and TiO_2_NPs/PrGO-based^22^ sensors detect NO_2_ at 10 ppm^21^ and 114 ppb^22^ under UV light, respectively. UV-activated CuO/TiO_2_^23^ and SnO_2_-based^19^ sensors detect H_2_S and CO at concentrations as low as 3 ppm and 100 ppb, respectively. Among MOx materials, TiO_2_ is particularly effective under UV light due to its excellent photocatalytic properties, especially for detecting gases like NO,^20^ NO_2_^21^, HCHO,^19^ and H_2_S.^23^ With a band gap of 3.2–3.4 eV,^18,19,24^ n-type TiO_2_ acts as an effective photocatalyst,^23^ significantly improving gas sensing performance. Notably, UV light with a wavelength of 365 to 370 nm has been shown to be particularly effective in exciting TiO_2_, significantly improving the gas sensing performance for NO_2_^21^ and H_2_S.^23^ Tin dioxide (SnO_2_), an n-type oxide known for its chemical stability, has a wider bandgap (3.6 to 4 eV)^19^ and benefits from photogenerated electrons supplied by TiO_2_, thereby improving gas-sensing capabilities. Overall, UV light facilitates adsorption and desorption reactions on MOx surfaces, enhances conductivity and charge transfer, and significantly improves sensor performance at RT.^25,26^

The role of UV-induced charge transfer occurring at MOx heterojunction interfaces particularly attracts our attention. In Table S1, the required UV intensity is usually in the range of mW/cm^2^. It is known that the penetration depth of UV light is very small; to greatly improve the UV catalyst efficiency, creating high-density heterojunctions on irradiating surfaces may be the key. The above research studies demonstrated that nanostructured heterojunctions^18−22^ can significantly enhance gas-sensing performance, while UV irradiation can effectively reduce the sensor’s operating temperature to RT. If considering simple fabrication, the prior reports mostly obtain the heterojunction by mixing nanoparticles, nanorods, and nanotubes into the sensing film. To clearly investigate the surface effect, we proposed a double-layer sensor structure by depositing sol–gel SnO_2_ onto a nanoporous TiO_2_ film. Reducing the thickness of the SnO_2_ layer to fill into the underlying nanopores, we successfully formed high-density SnO_2_/TiO_2_ nanoheterojunctions right on the irradiating surface. Note that the nonporous layered SnO_2_/TiO_2_ sensors acted as the control.

In this study, a unique nanoporous TiO_2_/SnO_2_ heterojunction matrix was developed for NO_*x*_ gas detection using a two-step sol–gel process. The TiO_2_ and SnO_2_ heterojunction matrix was formed on the film’s surface, allowing the TiO_2_ photocatalyst to readily absorb low UV power and efficiently transfer electrons to the SnO_2_ conduction band. Consequently, our proposed sensor requires very low UV power, around 3 μW/cm^2^, which is the minimum UV power needed to activate the photocatalytic effect in gas sensors compared to reported values in the literature (Table S1). The nanoporous TiO_2_/SnO_2_ sensor significantly enhances gas sensing performance while utilizing minimal UV power (3 μW/cm^2^), operating at 1 V to deliver a current higher than 10^–6^ A at RT. The remarkable sensing performances were obtained, including ppb-level detection of NO and NO_2_ gases (4 and 10 ppb, respectively), extended lifetime (∼30 days), rapid response and recovery times, enhanced stability, and increased response across a wide relative humidity (RH) range (10–60%). As a comparison, the nonporous thin-film TiO_2_/SnO_2_ requires high UV power (40 μW/cm^2^) to develop a stable sensor. Additionally, the ultralow UV power required can be easily harvested from sunlight, allowing the sensor to function effectively without the need for UV LEDs. Table S2(28−35) compares recent MOx-based NO gas sensors at low temperatures, covering fabrication methods, temperatures, electrode materials, gas response, and LOD, highlighting our sensor’s competitive LOD and power efficiency. Overall, the proposed nanoporous sensor with a SnO_2_/TiO_2_ heterojunction matrix provided a sustainable, cost-effective solution for NO_*x*_ detection. With a detection limit for NO gas (4 to 500 ppb), it is ideal for monitoring exhaled NO in asthma patients (25 to 50 ppb), making it suitable for clinical applications while ensuring patient safety with its low UV intensity.

## Experimental Procedure

### Materials

Tin(IV) chloride pentahydrate (SnCl_4_·5H_2_O) was purchased from Sigma-Aldrich, and ethanol from Honeywell was used to prepare the SnO_2_ solution. Methacrylic acid (Aldrich), titanium tetraisopropoxide (Aldrich), n-propanol (Choneye Pure Chemicals), and Pluronic F127 (Sigma-Aldrich) were used for the preparation of the TiO_2_ solution.

### Fabrication of the UV-Activated NO_*x*_ Gas Sensor and Measurement

**Figure 1:**
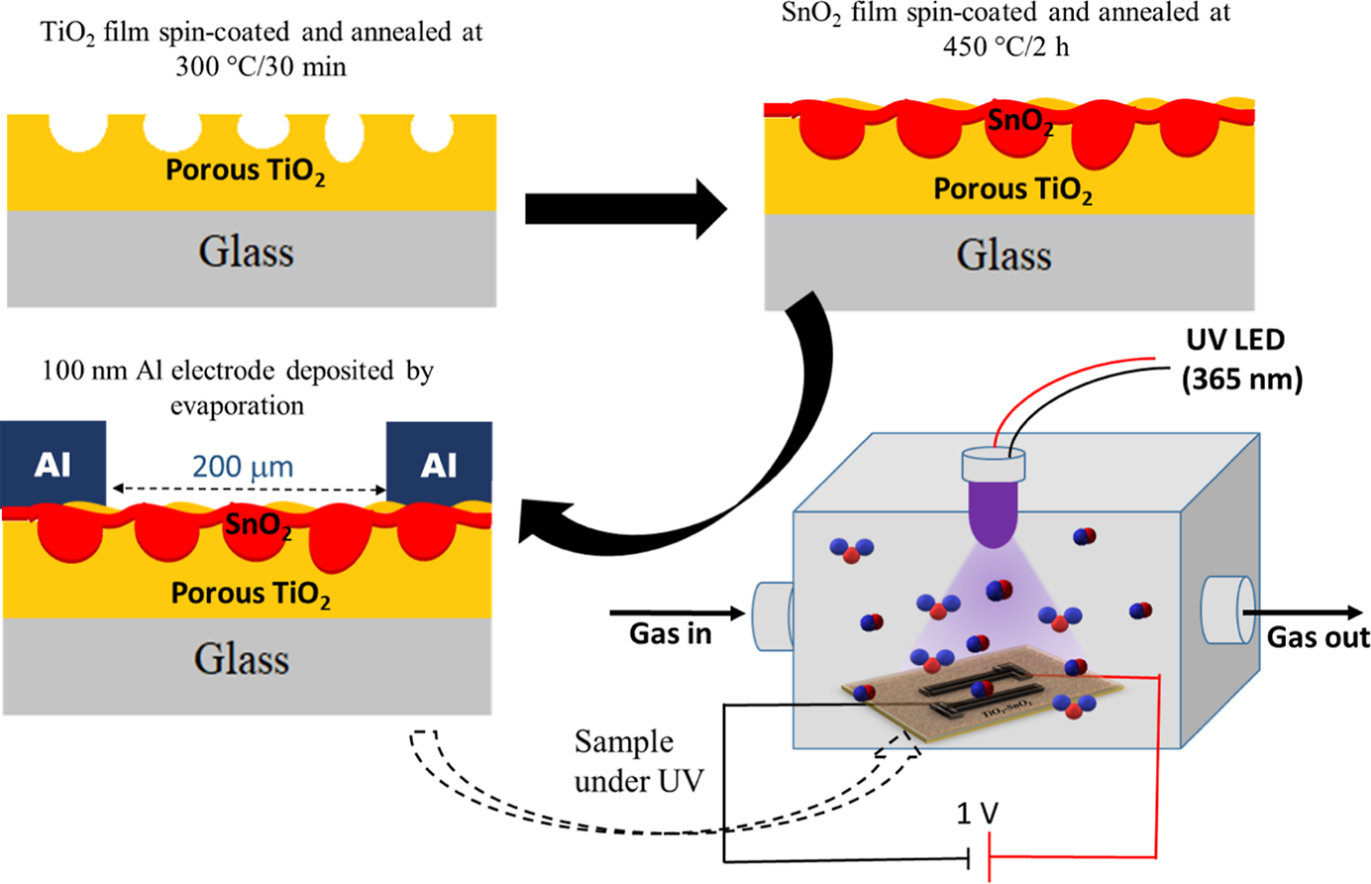
Fabrication of the porous TiO_2_/SnO_2_-based sensor and measurements under UV illumination.

Figure 1 depicts the sensor fabrication process and the UV-activated gas sensing setup. The porous TiO_2_/SnO_2_ sensor was produced through a two-step sol–gel process. In prior reports, porous structures were mostly formed by ALD,^24^ CVD,^36^ flame annealing,^37^ hydrothermal,^38^ and sol–gel^39^ techniques. The TiO_2_ synthesis process was adapted from previous works:^40^ dense films were obtained from formulations prepared with methacrylic acid (2 mL) added to titanium tetraisopropoxide (0.85 g) and stirred for 5 min. Then, n-propanol (2 mL) was introduced and stirred for 10 more minutes. Deionized water (1 mL) was added, and stirring was continued for 1 h. Porous TiO_2_ was obtained by adding pluronic F127 (0.0175 g) to the formulation. The solution was aged unstirred for 24 h. Before use, n-propanol (1.5 mL) was added, followed by 20 min of stirring. Spin-coating onto a glass substrate at 3000 rpm for 60 s was performed, followed by annealing at 300 °C for 30 min, yielding a TiO_2_ film of 65 ± 5 nm thickness. Porosity in the TiO_2_ thin film is obtained by adding pluronic F127 as a surfactant in the formulation. This method is simple and controllable with high reproducibility. For SnO_2_, a solution of 0.2 M SnCl_4_·5H_2_O in a 1:1 ethanol/DI water ratio was stirred for 24 h. Subsequently, the SnO_2_ precursor^41,42^ solution was spin-coated onto the porous TiO_2_ film at 2000 rpm for 60 s and then annealed at 450 °C for 2 h, producing a SnO_2_ film with a thickness of 30 ± 5 nm. Finally, 100 nm aluminum (Al) electrodes were deposited atop SnO_2_ to complete the sensor. Furthermore, the gas sensing measurements were conducted under UV illumination, utilizing a system detailed^42−44^ in the Supporting Information (Figures S1 and S2). Additionally, the fabrication procedure for pure SnO_2_ and nonporous TiO_2_/SnO_2_-based sensors is outlined in Figure S1.

## Results and Discussion

### Characterization

**Figure 2:**
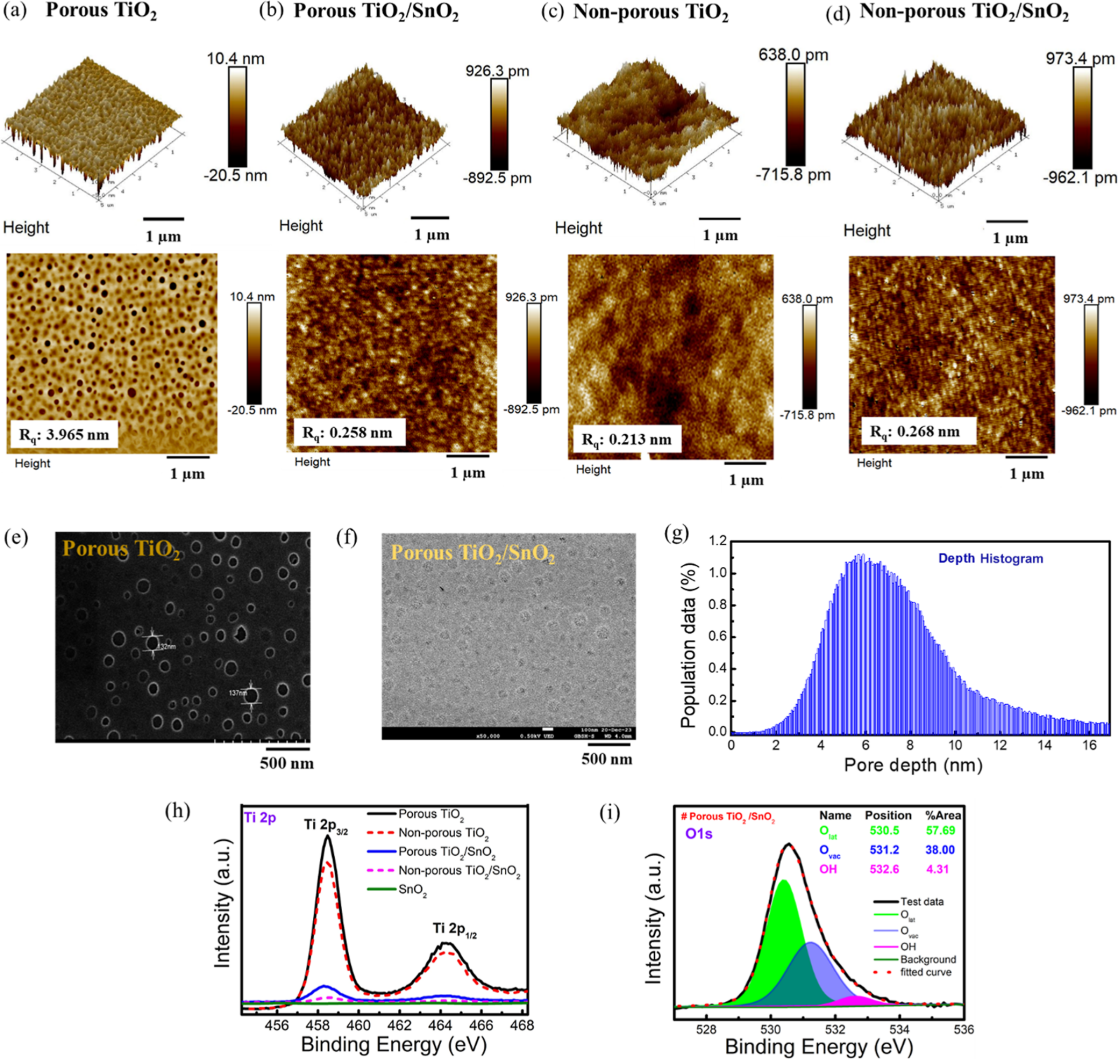
Morphological analysis of films. (a–d) AFM (5 × 5 μm^2^) images of porous TiO_2_, porous TiO_2_/SnO_2_, nonporous TiO_2_, and nonporous TiO_2_/SnO_2_. (e,f) SEM images of porous TiO_2_ and porous TiO_2_/SnO_2_, and (g) pore depth histogram images of porous TiO_2_ from the AFM (5 × 5 μm^2^) image. XPS images of (h) Ti-2p on five different films and (i) O 1s components of porous TiO_2_/SnO_2_ films.

The morphological characteristics of the films were analyzed by atomic force microscopy (AFM) and scanning electron microscopy (SEM). Figure 2a–d displays the 2D and 3D AFM (5 × 5 μm^2^) images of porous TiO_2_, porous TiO_2_/SnO_2_, nonporous TiO_2_, and nonporous TiO_2_/SnO_2_ films. Porous TiO_2_ exhibits higher surface roughness (*R*_q_ = 3.965 nm) with distinct nanoporous structures compared to nonporous TiO_2_ (*R*_q_ = 0.212 nm). The porous and nonporous TiO_2_/SnO_2_ films display similar surface roughness (*R*_q_) values of 0.258 and 0.268 nm, respectively, which reveals that both sensors may exhibit similar gas response capabilities. SEM images in Figure 2e,f confirm the presence of porous nanoporous structures in porous TiO_2_ and porous TiO_2_/SnO_2_ films, respectively, emphasizing the alignment of SnO_2_ on the porous TiO_2_. This alignment underscores the significance of the TiO_2_ and SnO_2_ interaction, which is crucial for our proposed gas sensing performance, as evident from the surface features observed in Figure 2f. Additionally, the depth histogram profile (Figure 2g) of TiO_2_ nanopores, extracted from the AFM image (Figure 2a), reveals that the majority of pore depths range between 5 and 8 nm.

We conducted energy-dispersive X-ray spectroscopy (EDX) analysis to examine the surface composition of the SnO_2_, porous, and nonporous TiO_2_/SnO_2_ films. The EDX spectrum confirms the presence of Sn, Ti, and O elements in the porous TiO_2_/SnO_2_ film and Si, C, Na, K, Al, and O elements from the glass substrate (Figure S3a,c,e). To address this, we conducted EDX mapping for each element across the film’s surface (Figure S3b,d,f), revealing a uniform distribution of all elements.

XPS analysis (Figure S4a) confirmed the chemical composition and state of all films containing TiO_2_ and SnO_2_. In both pure SnO_2_ and porous TiO_2_/SnO_2_, the binding energies^45^ of the Sn 3d_5/2_ and 3d_3/2_ peaks (Figure S4b) were observed at 486.3 and 494.8 eV, respectively. In contrast, for nonporous TiO_2_/SnO_2_, these peaks are located at 486.6 and 495.1 eV, respectively. The slight differences in binding energy values between porous and nonporous TiO_2_/SnO_2_ may be due to variations in the surface area, electronic interactions, and local chemical environments, which affect the electronic states and bonding of atoms. The difference between the two Sn 3d peaks is 8.5 eV, indicating a spin–orbit splitting of 8.5 eV (Figure S4b), which confirms the Sn^4+^ oxidation state in both films.^46^ Symmetrical peaks^46^ of Ti 2p, representing 2p_3/2_ and 2p_1/2_ at 458.5 and 464.3 eV respectively, indicate a 5.8 eV difference, signifying the valence state of Ti 2p as +4 (Figure 2h). Notably, the Ti intensity on the surface of porous TiO_2_/SnO_2_ film is higher than nonporous TiO_2_/SnO_2_, verifying the high-density heterojunctions on the surface to enhance the photocatalytic effect of the gas sensor. The O 1s peaks consist of three components: lattice oxygen (O_lat_) at 530.3 ± 0.3 eV, oxygen vacancy (O_vac_) at 531.1 ± 0.1 eV, and hydroxyl group (OH) at 532.5 ± 0.1 eV (Figure 2i). Among these, O_vac_ plays a vital role in gas sensing performance, which will be discussed in detail in the mechanism section. Raw XPS data for SnO_2_ (Figure S4c) and nonporous TiO_2_/SnO_2_ (Figure S4d) are provided in the Supporting Information. UV–vis absorption spectra (Figure S5) revealed band gaps of 3.9 eV for pure SnO_2_ and 3.3 eV for porous and nonporous TiO_2_, while both porous and nonporous TiO_2/_SnO_2_ showed a similar band gap of 3.63 eV. Consequently, a 365 nm UV LED was considered sufficient to excite TiO_2_ in the TiO_2_/SnO_2_ structure for subsequent gas sensing measurements. In the following section, our study focuses on the gas sensing performance of the sensor.

### Assessment of Crucial Parameters for NO_*x*_ Gas Sensing Performance

**Figure 3:**
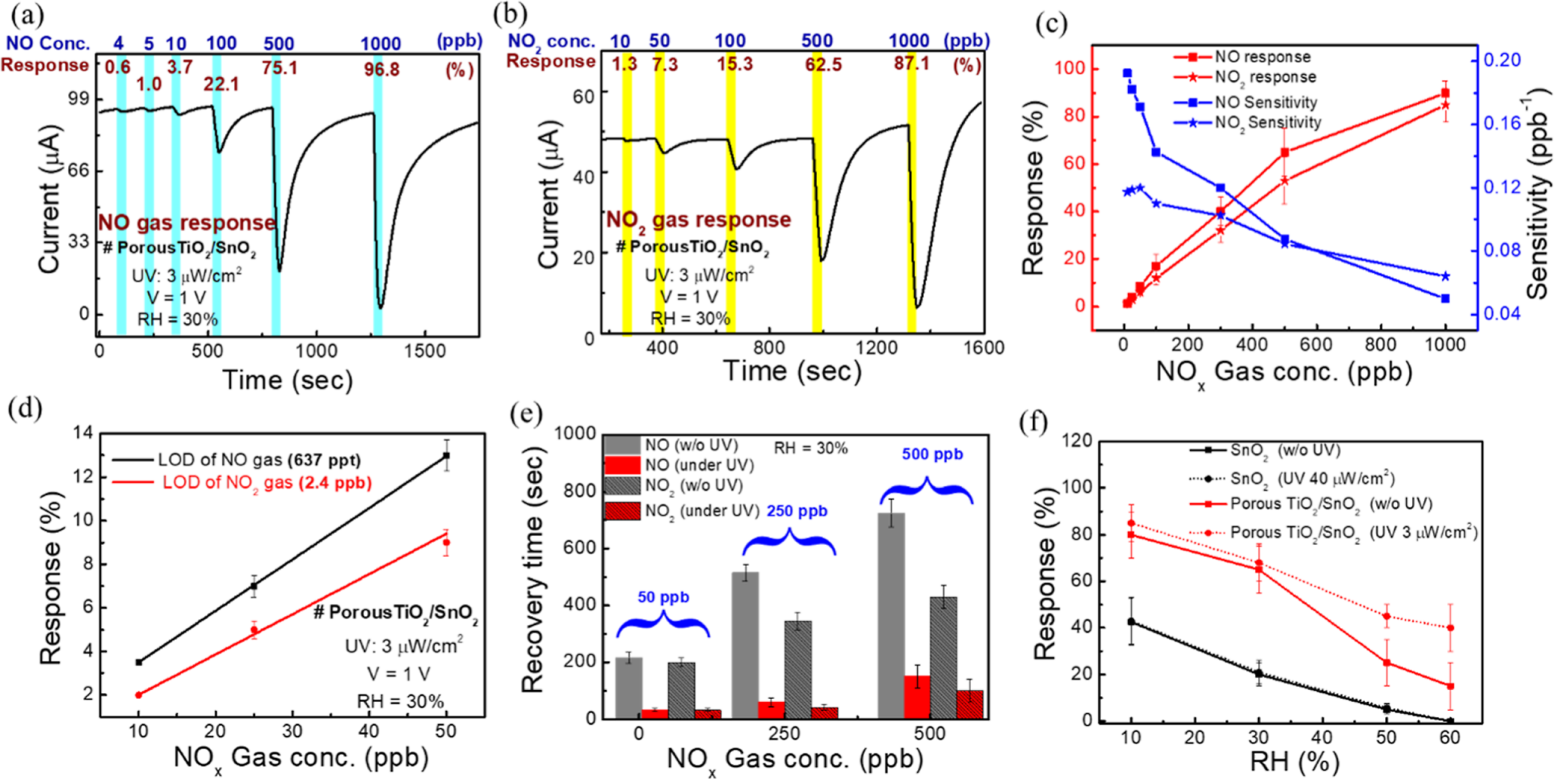
Gas sensing data of the porous TiO_2_/SnO_2_-based sensor under 365 nm (3 μW/cm^2^) UV illumination, (a) NO and (b) NO_2_ dynamic response data. (c) Gas response and sensitivity calibration curves under different NO and NO_2_ gas concentrations. (d) Detection of the LOD for NO_*x*_ gas. (e) Recovery time data at different NO_*x*_ gas concentrations under UV and without UV illumination. (f) Relative humidity effect test: effect of RH on the gas response of different sensors, including pure SnO_2_ and porous TiO_2_/SnO_2_, under UV and without UV illumination at 500 ppb NO gas concentration.

In this section, crucial parameters of the gas sensor were investigated, including gas response, sensitivity, LOD, and response/recovery time of the porous TiO_2_/SnO_2_-based sensor operating at a low voltage of 1 V under 30% RH at room temperature. The I–V curve (Figure S6a) of the sensor under UV (3 μW/cm^2^) and without (w/o) UV illumination demonstrates ohmic behavior, with the sensor exhibiting high current levels (microamperes) at low voltages such as 1 V. Hence, we opted to maintain the operating voltage at 1 V for further experiments. To assess the dynamic response of the NO_*x*_ sensor, it was analyzed under different NO concentrations ranging from 4 to 1000 ppb (Figure 3a) and NO_2_ concentrations from 10 to 1000 ppb (Figure 3b), with a constant injection time set at 30 s. The responses of NO and NO_2_ at the lowest gas concentrations are 0.6% (at 4 ppb) and 1.3% (at 10 ppb), respectively. The real-time NO_*x*_ sensing data w/o UV illumination indicate that the sensor either fails to fully recover (Figure S6b) or exhibits slow recovery (Figure S6b,c). Notably, in Figure S7, the pure SnO_2_-based sensor does not show any improvement in recovery under UV (Figure S7b), whereas the nonporous TiO_2_/SnO_2_-based sensor demonstrates good recovery under UV illumination at 40 μW/cm^2^ (Figure S7c). The real-time gas dynamic NO_*x*_ gas sensing data’s capability to provide immediate responses to changing environmental conditions ensures accurate monitoring in the ultra ppb region at room temperature. Additionally, Figure 3c, with double *y*-axis data, presents calibration curves of response and sensitivity, providing insight into the sensor’s applicability in the 10 to 1000 ppb NO_*x*_ gas region with error bars (3 samples). This data suggests the sensor’s suitability for concentrations below 1000 ppb. The response versus gas concentration data depicts the sensor’s linear range between 10 and 1000 ppb, making it suitable for environmental monitoring. The response is calculated as the ratio of the change in current (*I*_bg_ – *I*_gas_) to the original background current (*I*_bg_).^42,43^ Here, *I*_gas_ represents the current in the presence of a gas.
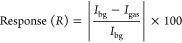
1

Furthermore, to gain a better understanding of the specific region of applicability, sensitivity curves were plotted, indicating the change in response (*R*) with respect to gas concentration (c), i.e., d*R*/d*c*.^42,47^ The sensitivity of the NO gas sensor increases with decreasing NO concentration from 1000 to 100 ppb, followed by a sharp increase after 100 ppb, indicating higher sensitivity at lower concentrations. This suggests the sensor’s suitability for breath NO detection in asthma^4^ patients, where low NO gas detection (<100 ppb) is required. Conversely, sensitivity increases slowly with decreasing concentration from 1000 to 100 ppb for NO_2_, becoming constant below 100 ppb. Hence, this sensor should be applicable for environmental monitoring. Notably, the sensitivity of NO is higher than that of NO_2_ in the 10 to 500 ppb region, making this sensor more preferable for NO detection compared to NO_2_ at low concentrations (<500 ppb).

Based on real-time gas sensing measurements (Figure 3a,b), we have observed experimental LOD for NO and NO_2_ gases at 4 and 10 ppb, respectively. Consequently, our focus is on determining the theoretically calculated LOD value from the fitted linear region of NO_*x*_ gas at low concentrations (10, 25, and 50 ppb). The LOD value was defined as 3 times the ratio of the standard deviation (Sy) to the slope (*S*) of the calibration curve, i.e., 3(Sy/*S*).^42,48^ The fitted calibration curve demonstrates good linearity with *R*^2^ values of 0.99989 and 0.99255 for NO and NO_2_ gases, respectively, resulting in LOD values of 637 parts per trillion (ppt) and 2.4 ppb (Figures 3d; S2 and eq S1).

The response/recovery time (*T*_90_) in MOx-based gas sensors is slow at room temperature, according to the literature^28−35^ (Table S2). Hence, improving recovery time using ultralow UV power intensity (3 μW/cm^2^) is crucial. Figure 3f reveals a 7-fold decrease in recovery times of NO and NO_2_ after UV illumination compared to without UV. The recovery time for 50 and 500 ppb NO is 33 ± 5 s and 150 ± 50 s and that for NO_2_ is 33 ± 5 s and 100 ± 40 s, respectively. Thus, low UV power intensity effectively improves the recovery time of porous TiO_2_/SnO_2_-based NO_*x*_ sensors. After UV illumination, photocatalytic TiO_2_ absorbs UV light, initiating electron–hole pair generation. The combination of TiO_2_’s photocatalytic activity under UV and NO_*x*_ molecule oxidation leads to faster recovery of TiO_2_/SnO_2_-based NO_*x*_ sensors. Enhanced desorption of the oxidized species from the sensor surface contributes to accelerated recovery. Additionally, the sensor was measured with a 30 s injection time, defining the response time (*T*_90_) as 30 s.

To understand the effect of RH on NO sensors, we measured the performance of an optimized porous TiO_2_/SnO_2_ sensor under both optimized UV illumination and without UV exposure, comparing these results with an optimized pure SnO_2_-based sensor. The response vs RH curve (Figure 3f) at a 500 ppb NO concentration over RH levels ranging from 10% to 60% shows that the incorporation of the porous TiO_2_ structure enhances the sensor response compared to pure SnO_2_. This improvement may be attributed to the nanoheterojunction matrix, which potentially increases the surface active sites, facilitating greater gas molecule adsorption. Notably, the results indicate a decrease in sensor response with increasing RH levels. In low RH, the limited amount of water molecules does not significantly affect the sensor’s response. As RH increases, water molecules attach to the sensor and compete with NO gas molecules, reducing the active sites available for NO gas molecules. Consequently, increasing RH decreases the sensor’s response^19^ (Figure S8a–f). However, it is observed that the porous structure^27^ and UV illumination help to improve the response at high RH. Porous TiO_2_/SnO_2_-based sensors show an improvement in response at high RH under UV illumination, whereas the SnO_2_-based sensor does not exhibit any improvement in response under UV illumination. The dynamic sensing response data and response vs gas concentration calibration plots for both sensor types are provided in the Supporting Information (Figure S8a–d). These plots further illustrate the enhanced performance of UV-activated porous TiO_2_/SnO_2_-based sensors under varying RH conditions compared to pure SnO_2_ sensors. Under UV illumination, the photogenerated electrons from TiO_2_ improve the response of the TiO_2_/SnO_2_-based sensor, indicating the availability of more NO active sites. Figure S9a shows the UV-activated real-time data of the porous TiO_2_/SnO_2_-based sensor, demonstrating its dynamic response to 500 ppb of NO across 10–60% RH, highlighting its versatility. Figure S9b reveals that this sensor is capable of detecting both NO and NO_2_ gas in the low (50 ppb) to high (500 ppb) ppb range in different RH. Overall, this section clarifies that the proposed UV-activated NO_*x*_ gas sensor shows promising results in terms of response, sensitivity, LOD, response/recovery time, and endurance under different RH. Furthermore, the next section will focus on the importance of UV effects on porous TiO_2_/SnO_2_-based NO gas sensors in stabilizing current, response, and recovery performance.

### Importance of UV Illumination to Stabilize the Sensor

**Figure 4:**
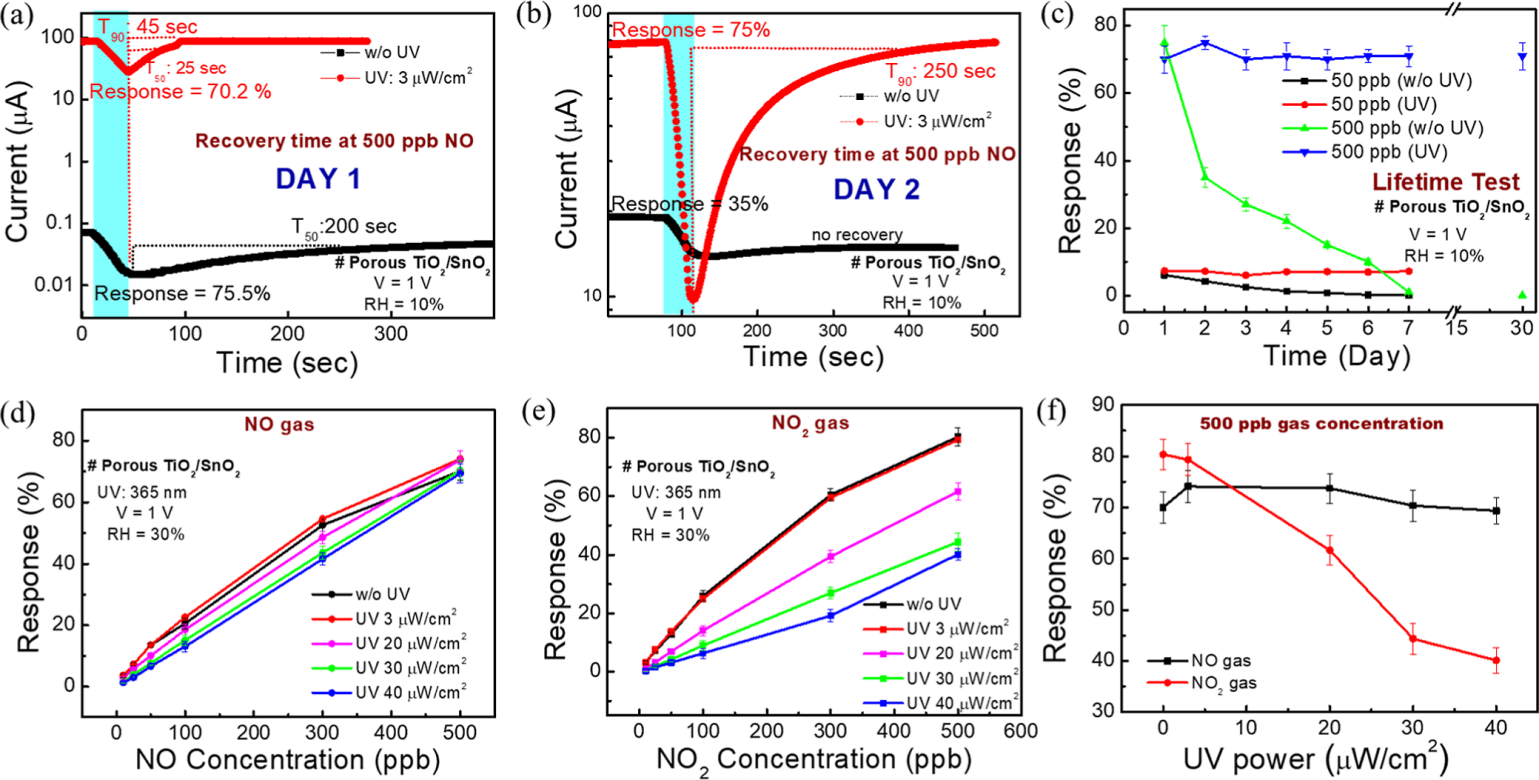
Importance of UV illumination for sensor stabilization shown by comparing gas response data on the porous TiO_2_/SnO_2_-based sensor over (a) day 1, (b) day 2, and (c) up to 30 days under UV and without UV illumination. UV-power effect of the NO and NO_2_ sensor: (d,e) response vs NO and NO_2_ gas concentration calibration curves of the porous TiO_2_/SnO_2_-based sensor under different UV powers. (f) Response vs UV power calibration curve at a 500 ppb NO and NO_2_ gas concentration.

UV illumination plays a crucial role in stabilizing the sensor by maintaining a constant current level, response, and full recovery. Without UV illumination, the sensor exhibits unstable current levels, a degraded response, and recovery issues by the second day. As shown in Figures S10a–c, there is a drastic increase in current levels with rising RH without UV illumination on day 1 but no significant change in current levels under different RH conditions on day 2 without UV illumination. In contrast, under UV illumination, the current level remains constant and stable, indicating that water molecules have less effect on the current level due to continuously photogenerated electrons, which help maintain a stable current. Furthermore, the calibration curve (Figure S10d) of the gas response at different NO concentrations shows a stable response on both day 1 and day 2 under UV illumination. Without UV illumination, the response decreases to 50% of its original value on day 2. This decrease may be attributed to reduced oxygen vacancies on day 2 without UV conditions. Without UV, the attachment of water molecules on the surface of SnO_2_ may increase, while UV illumination helps dissolve water molecules and restore adsorption sites. Figure 4a,b illustrates the effect of UV light on sensor recovery and recovery speed on days 1 and 2. This experiment is typically conducted at low RH levels, such as 10%, because the recovery issue becomes more pronounced as the RH decreases. Notably, on day 1, the sensor exhibits only 50% recovery, and there is no recovery on day 2 under 500 ppb of NO without UV illumination. However, the sensor demonstrates full recovery on both days 1 and 2 with recovery times (*T*_90_) of 45 and 200 s, respectively. Figure S10e,f depicts the relationship between the recovery ratio (S3 and eq S2) and its original position under different RH conditions at low (50 ppb) and high (500 ppb) gas concentrations. Over a period of 30 days, it is evident that the sensor shows a stable NO response under UV illumination compared to that without UV illumination (Figure 4c). Thus, the sensor exhibits full recovery under UV in various humid conditions over time, contributing to its stability. The UV-activated porous TiO_2_/SnO_2_-based NO_*x*_ sensor will be further used to understand the effect of UV power on the sensor in the next section due to its superior performance compared to that of the other two sensors.

### UV Power Effect on NO and NO_2_ Gas Response

Characterizing the UV effect on gas sensors is crucial, especially for environmental monitoring. Sunlight UV can impact sensors used for environmental gas monitoring, making it necessary to assess the effect of UV on NO_*x*_ sensors. Hence, research has focused on detecting sensor responses at various UV intensities. Figure 4d,e illustrates the response versus gas concentration calibration curve of porous TiO_2_/SnO_2_-based NO and NO_2_ gases under different UV intensities, respectively. A minimal decrease in NO response (with a relatively constant response) is observed as the UV intensity increases from 3 to 40 μW/cm^2^ (Figure 4d). Conversely, the response of NO_2_ significantly decreases with rising UV intensity (Figure 4e). Additionally, Figure 4f clearly shows a 50% drop in response at 40 μW/cm^2^ compared to the original optimal response at 3 μW/cm^2^ for 500 ppb of NO_2_, whereas the response drop for NO is only 6%. The influence of UV intensity on the gas response may be inferred from the absorption spectra of NO and NO_2_ gases. According to the literature, the UV absorption peaks^49,50^ for NO are between 220 and 280 nm, while NO_2_ shows absorption peaks^50,51^ between 370 and 400 nm. In our work, we used 365 nm UV light. Thus, NO_2_ gas reacts directly with the UV light before reaching the TiO_2_/SnO_2_ sensing material, reducing the NO_2_ response. In contrast, the NO absorption spectrum peak is far from the UV illumination used. Therefore, the NO response remains unaffected by UV light intensity, whereas the NO_2_ response is influenced by the UV power intensity (Figure 4f). Based on this discussion, it can be concluded that our proposed NO sensor performs well across various environmental conditions. Therefore, our subsequent discussions, such as reliability testing and comparison of the proposed sensor with existing literature, will primarily focus on porous TiO_2_/SnO_2_-based NO sensors.

### Reliability Test

**Figure 5:**
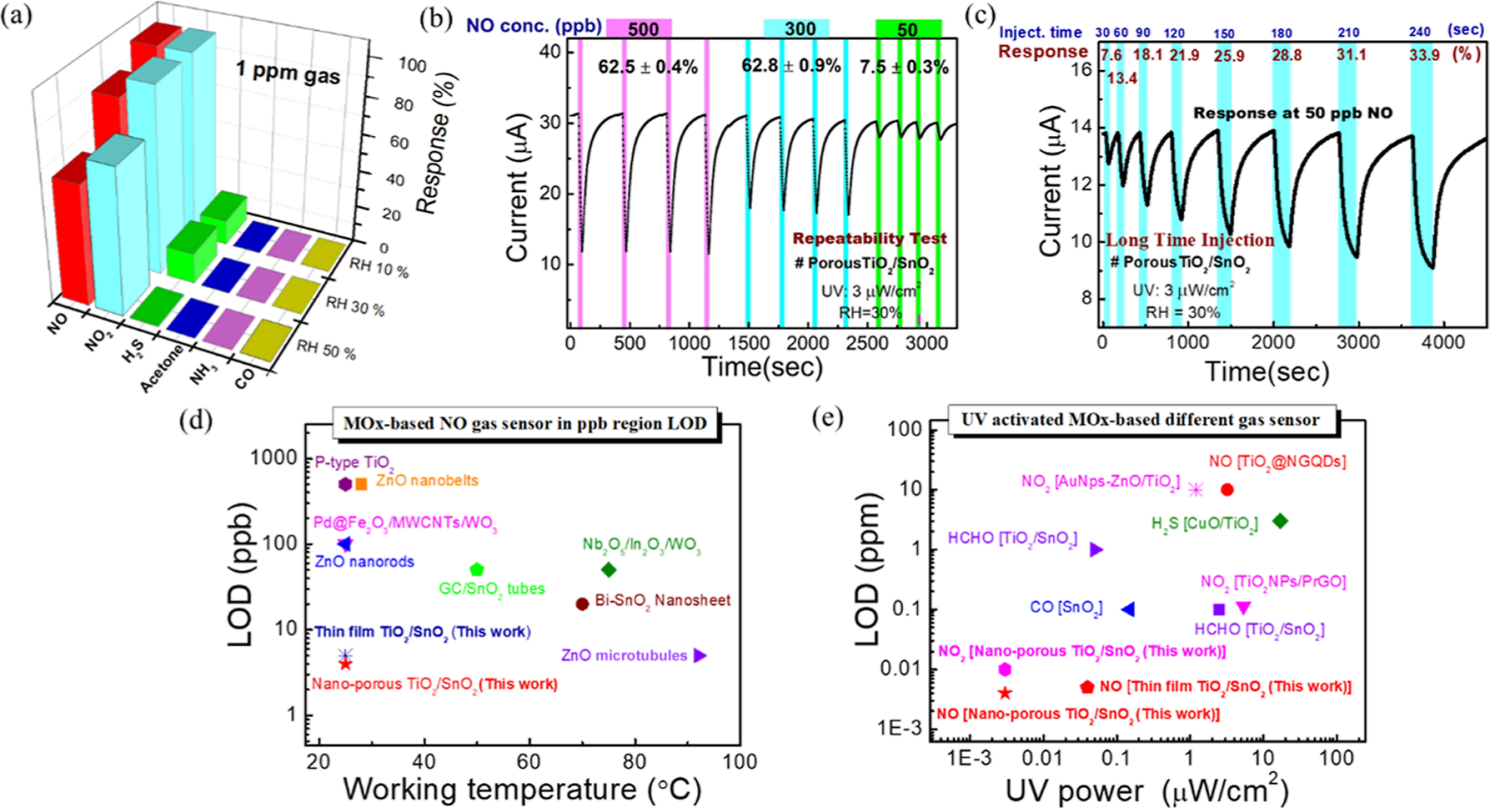
Reliability test of the porous TiO_2_/SnO_2_-based sensor. (a) Selectivity test for 1 ppm of different gases at varying RH levels. (b) Repeatability data over four cycles for NO concentrations of 50, 300, and 500 ppb under UV illumination. (c) Detection data for 50 ppb NO gas at different injection times (30 to 240 s) to assess long-term detectability. (d) Overall performance comparison^28−34^ of the proposed TiO_2_/SnO_2_-based NO sensor with the best low LOD of NO sensors (MOx-based). (e) Comparison^18−23^ of MOx-based low-power UV-activated gas sensors with their LOD at room temperature.

Ensuring sensor reliability for practical applications necessitates conducting reliability tests, including assessing selectivity, repeatability, long-term detectability, and lifetime. Selectivity was evaluated by exposing the sensor to 1 ppm of various oxidizing and reducing gases (e.g., NO, NO_2_, H_2_S, NH_3_, acetone, CO) at different RH levels. The 3D selectivity curve (Figure 5a) demonstrates the sensor’s high selectivity toward NO and NO_2_ across varying relative humidities compared to other gases. Remarkably, it exhibits limited sensitivity to H_2_S (17%) at low RH (<30%) and no response at high RH, while showing no response to other gases (Figures 5a and S11). The sensor also showed no response to 500 ppm of CO_2_ gas (Figure S11d). Hence, we conclude that the proposed sensor exhibits excellent selectivity toward NO_*x*_. The cross-sensitivity^52,53^ between NO and NO_2_ can be addressed by using a calibration curve of sensor response versus gas concentration. The experiment aimed to assess the sensor’s repeatability under varying gas concentrations of 50, 300, and 500 ppb (Figure 5b). Results indicate consistent responses of 7.5 ± 0.3%, 42.8 ± 0.9%, and 62.5 ± 0.4% over four consecutive cycles, demonstrating excellent repeatability. To understand long-term durability, the sensor was tested with different injection times ranging from 30 to 240 s at a constant gas concentration of 50 ppb. It is notable that the sensor is able to fully recover after long-time gas injection. Figure 4c depicts the sensor’s lifetime with and without UV illumination at concentrations of 50 and 500 ppb over a 30 day period at RH 10%. Without UV, the sensor’s response decreases daily (Figure S11b), while under UV (3 μW/cm^2^), it maintains stability throughout (Figure S11c). UV light aids in oxygen adsorption and reactivates active sites on the sensor surface, countering the reduction of oxygen vacancies. This discussion confirms the reliability of the UV-activated porous TiO_2_/SnO_2_-based NO_*x*_ sensor for future applications. Notably, the pure SnO_2_-based sensor exhibits instability under UV exposure (Figure S12a), while the nonporous TiO_2_/SnO_2_ thin film-based NO sensor shows an unstable response both without UV (Figure S12d) and under low UV power (3 μW/cm^2^) (Figure S12e), with performance degrading over time. However, at a higher power (40 μW/cm^2^), the sensor maintains a stable response over 7 days (Figure S12f). Real-time NO gas response data at 500 ppb using the porous TiO_2_/SnO_2_ sensor shows 65.3% on day 1 (Figure S12g) and 62.5% on day 30 (Figure S12h) under 3 μW/cm^2^ UV illumination, demonstrating stable performance over 30 days at RH 30%. Additionally, the response at RH 10% is 71.0% on day 30 (Figure S12i), similar to day 1 (Figure 4a). Overall, both porous and nonporous TiO_2_/SnO_2_-based sensors show a good lifetime under UV illumination (Figure S12c,f). The nonporous sensor requires higher UV power due to TiO_2_’s photocatalytic effect on the SnO_2_ surface. Conversely, the porous TiO_2_ structure necessitates lower UV power as SnO_2_ resides within TiO_2_ pores, enabling effective photocatalysis at the SnO_2_ surface. Based on these discussions, we can conclude that the sensor is reliable for practical applications.

### Comparative Analysis of the Proposed Sensor with the Literature

To comprehend the novelty of our work, we provide a competitive graphical analysis of the literature, focusing particularly on low power consumption and low LOD for gas detection. Figure 5d illustrates the LOD of MOx-based NO gas sensors at low working power in the ppb region (data from Table S2).^28−35^ The LOD vs working temperature curve reveals that both our proposed porous and nonporous TiO_2_/SnO_2_-based NO sensors exhibit the lowest LOD, at 4 and 5 ppb, respectively, at room temperature (24 ± 1 °C). Additionally, Figure 5e demonstrates UV-activated (low-powered) gas sensors at room temperature, where our proposed NO_*x*_ sensors also exhibit the lowest UV illumination compared to any other sensors in the literature (data from Table S1).^18−23^ In summary, our proposed NO_*x*_ sensor effectively reduces power consumption, addressing a significant drawback of MOx-based gas sensors, and enables the detection of NO_*x*_ gas in the ultra-ppb region at room temperature.

### Lifetime Test of NO_*x*_ Sensor under Sunlight

**Figure 6:**
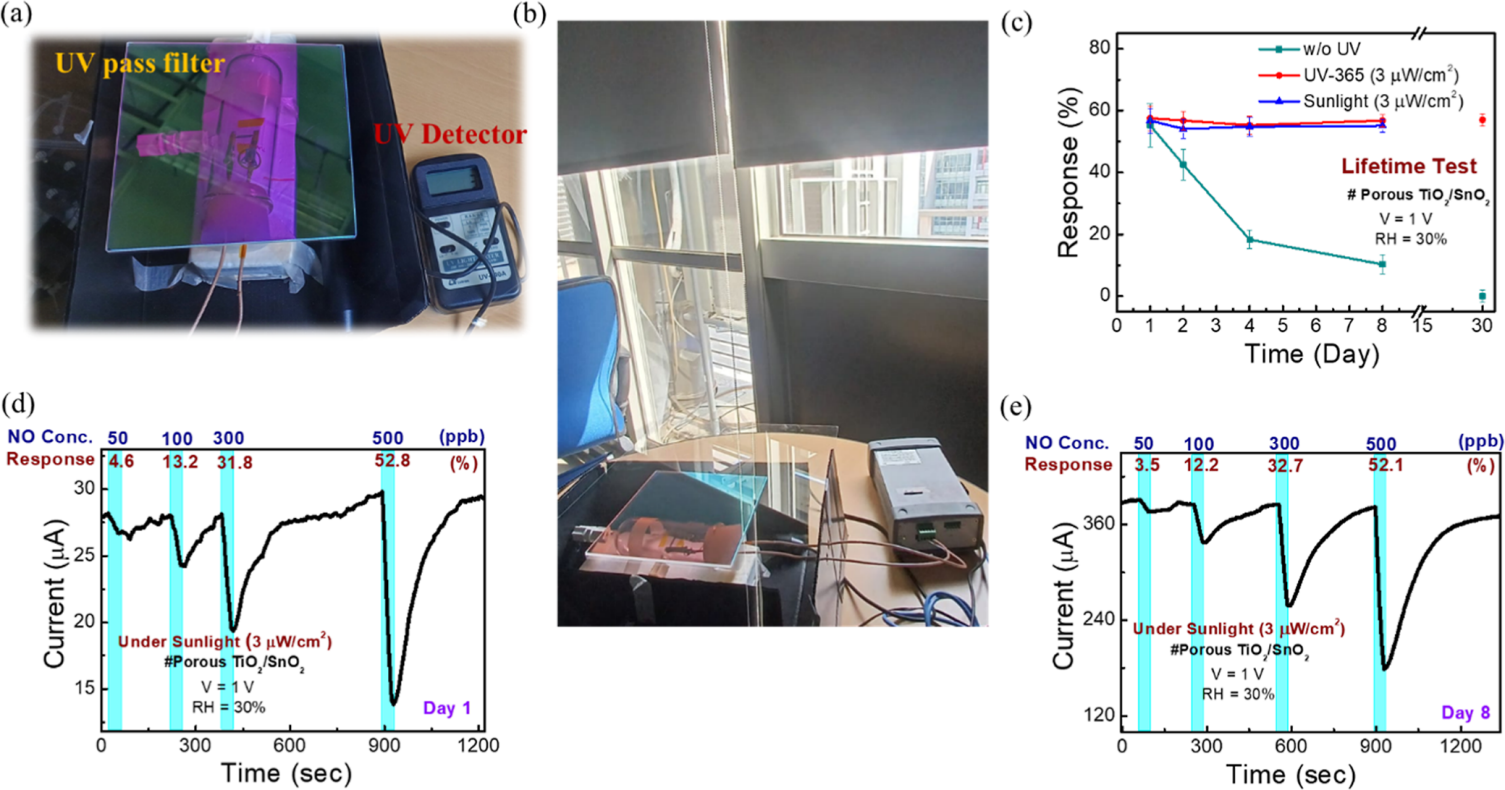
Lifetime test of the sensor under sunlight. (a) Image of the sensing chamber exposed to sunlight. (b) Setup of the sensing measurement system placed indoors with sunlight entering through a window. (c) Comparison of lifetime test data for 500 ppb NO sensing without UV, under UV, and under sunlight for the proposed sensor after 8 days and 30 days. (d) Real-time NO gas sensing data from 50 to 500 ppb under sunlight on day 1 and (e) day 8.

As discussed earlier, our proposed sensor requires minimal UV power (3 μW/cm^2^) to improve the sensor’s lifetime. Therefore, we aimed to evaluate the NO_*x*_ responses using low UV power harvested from natural sunlight. The UV power intensity was regulated by adjusting the window curtains, allowing for controlled amounts of sunlight to enter the room. We began by measuring the UV intensity under sunlight using a UV detector (UV340 A), which detects light in the 290–390 nm range. The detected sunlight intensity indoors, through a window, ranged from 1 to 20 μW/cm^2^, making it feasible to achieve stable gas sensing without external UV LEDs. To ensure selective UV transmission, we employed a UV pass quartz filter (340–450 nm) with 90% efficiency. Figure 6a shows the sensing chamber setup incorporating the UV filter and detector. The overall gas sensing setup is similar to that in Figure S2, with the sunlight intensity controlled at 3 μW/cm^2^. The system was positioned near a window (Figure 6b), where sunlight entered the room. To evaluate the effect of sunlight on sensor performance, we compared the lifetime test data for 500 ppb of NO sensing without UV, under 365 nm UV, and under sunlight for 8 days (Figure 6c). We included day 30 lifetime data points with and without UV illumination in Figure 6c, revealing that the sensor maintains a similar response up to 30 days under UV illumination. The results show that the sensor’s response under sunlight remains consistent, similar to the 365 nm UV (3 μW/cm^2^) data, while the response without UV decreases daily. Real-time NO sensing data from day 1 and day 8 for 50–500 ppb concentrations are shown in Figure 6d,e, demonstrating that the sensor maintains good response and full recovery under sunlight even after 8 days. Additional data in Figure S13, including *I*–*V* characteristics (Figure S13a) and the real-time dynamic response of the sensor to 300 ppb of NO gas under varying sunlight intensities (Figure S13b), reveal that the response increases with higher sunlight intensity. Although this data is preliminary, further calibration is necessary for future outdoor monitoring under different power intensities. The NO/NO_2_ sensing results (Figure S13c–f) also demonstrate that sunlight can enhance the sensor’s lifespan. In this section, we conducted preliminary tests under natural sunlight with a low UV intensity of 3 μW/cm^2^, demonstrating that the UV-enhanced prolonged lifetime of the proposed sensor may be realized using natural sunlight without using an additional UV LED.

Based on our study, the sensor’s current response (Figure 6d,e) tends to increase over time when exposed to sunlight, likely due to the wide range of UV filters (340–450 nm). In contrast, a stable current was maintained over 30 days under 365 nm UV illumination (Figure S12g,h). It is possible that the increase of the tail state or interface defects in the SnO_2_ film or at the SnO_2_/TiO_2_ interface refers to the absorption edge in the absorption spectra (Figure S5a), absorbing 340–360 nm light to produce photocurrent. In contrast, the 365 nm UV LED is mostly absorbed by TiO_2_, inducing a catalytic effect rather than generating a photocurrent in SnO_2_. In future, introducing the band-pass filter (360–380 nm) shall improve the current stability. Also, referring to Figure 4e, the response to NO_2_ varies under different UV intensities. If the proposed system is used to detect NO_2_, the calibration curves under various sunlight intensities (filtered with 360–380 nm band-pass filter) need to be established, and the UV intensity needs to be detected.

### Sensing Mechanism

TiO_2_/SnO_2_ sensors have gained attention for NO_*x*_ detection due to their improved recovery and stability under UV activation. In this system, SnO_2_ is the gas sensing element, while TiO_2_ serves as a photocatalyst, improving the overall sensor performance.

One key challenge is the recovery time at room temperature, but our sensor achieves full recovery with improved speed. UV-activated TiO_2_ generates electron–hole pairs (eq 2), enhancing photocatalytic activity, which accelerates gas desorption and improves recovery. The generated holes (h^+^) help release adsorbed oxygen species (eq 4), quickly resetting the sensor surface.

Under UV light, a dynamic balance between adsorption (eq 3) and desorption (eq 4) minimizes surface degradation, extending the sensor lifespan. Without UV, oxygen vacancies in the TiO_2_/SnO_2_ film decrease, reducing the gas response. XPS analysis confirmed a drop in oxygen vacancies from 39.40% (Figure S14a) to 26.74% (Figure S14b) after 7 days without UV. However, we could not track improvements in oxygen vacancies under UV due to the lack of an XPS setup for such conditions.

The interaction of photogenerated electrons and environmental oxygen with reacting gases NO and NO_2_ is discussed below. When UV light activates the sensor, it excites electrons in TiO_2_, promoting them to the conduction band and leaving behind holes in the valence band
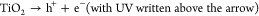
2

In the next equations, hν indicates the photon energy that is responsible for generating the electrons (e^–^) and hole (h^+^) through a photoexcitation process. The photogenerated electrons (e^–^) interact with adsorbed oxygen molecules, forming reactive oxygen species
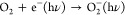
3

The generated holes (h^+^) contribute to the desorption of oxygen species
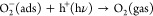
4

NO gas molecules react with photogenerated electrons, forming negatively charged NO species^20^
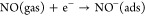
5

These NO further interact with adsorbed oxygen ions, leading to the formation of nitrogen species and oxygen vacancies on the sensor surface
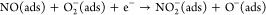
6
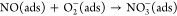
7

NO_2_ gas molecules interact with photogenerated electrons,^54,55^ forming NO_2_^–^ species
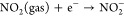
8

These NO_2_ can react with adsorbed oxygen species, leading to various nitrogen oxides^54^
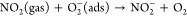
9
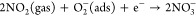
10

To verify the mechanism concept, we conducted XPS analysis on the samples before and after the injection of NO and NO_2_ gases. A significant N 1s peak^35^ at 400 eV, corresponding to the binding energy of nitrogen in NO_*x*_^–^ (*x* = 2 or 3), was clearly observed. This illustrates the oxidation of NO and NO_2_ to NO_*x*_^–^ by surface-adsorbed oxygen species.

**Figure 7:**
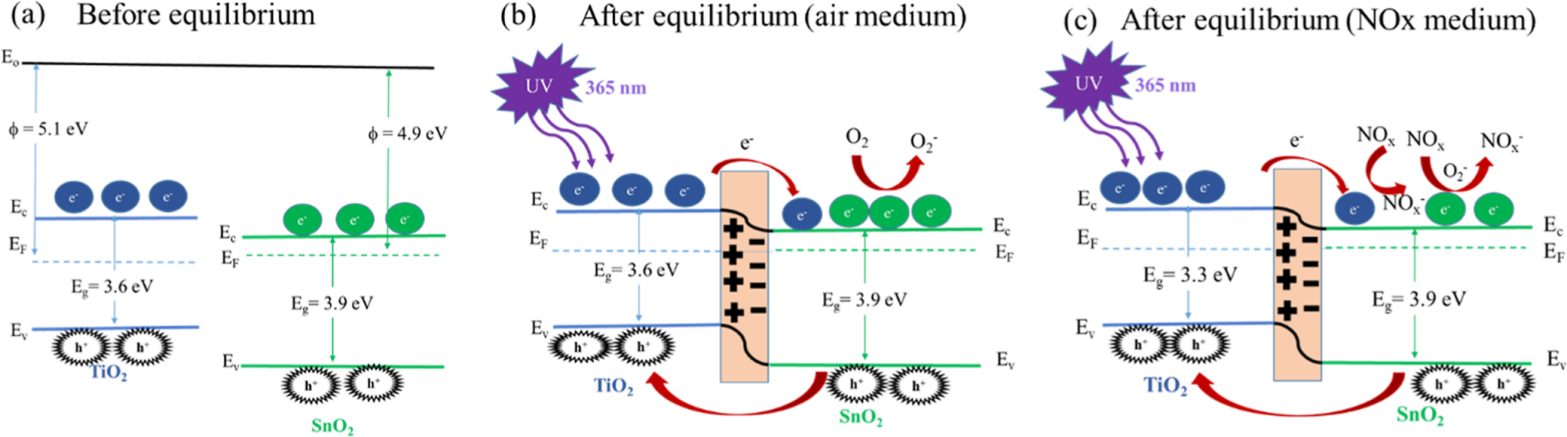
Energy band diagram of a TiO_2_/SnO_2_-based NO_*x*_ sensor: (a) before equilibrium, (b) under UV illumination in an air medium after equilibrium, and (c) in a NO_*x*_ medium.

The impact of UV on TiO_2_/SnO_2_ is significant, necessitating a focus on the band diagram of TiO_2_/SnO_2_. The electron transfer between the materials depends on their work functions. According to the literature,^24^ the work functions (ϕ) of TiO_2_ and SnO_2_ are 5.1 and 4.9 eV, respectively, before equilibrium (Figure 7a). This difference drives electron transfer from TiO_2_ to SnO_2_ until their Fermi levels align at equilibrium (Figure 7b). At equilibrium, a depletion region forms at the TiO_2_/SnO_2_ interface, halting electron transfer in the absence of UV illumination. Upon UV illumination, the free electron density on the TiO_2_ surface increases, enabling electron transfer to the SnO_2_ conduction band, which increases the current. Concurrently, environmental O_2_ molecules adsorb onto the SnO_2_ surface, capturing electrons from the conduction band and forming O_2_^–^. When NO_*x*_ gas is introduced, NO_*x*_ molecules capture O_2_^–^ and electrons from the SnO_2_ surface, resulting in increased resistance and a corresponding current drop (Figure 7c). Upon removal of NO_*x*_, the captured electrons return to the SnO_2_ conduction band, restoring the baseline current. Overall, under UV illumination, the continuous generation of electron–hole pairs improves gas sensing performance, including recovery, stability, cross-humidity effects, and sensor lifetime.

In this research, we identified key factors contributing to the performance of TiO_2_/SnO_2_-based NO_*x*_ gas sensors operating at room temperature, including:

(i) Enhanced sensor stability through the photocatalytic effect of TiO_2_ on the TiO_2_/SnO_2_ surface.
(ii) Activation of the photocatalytic effect in the gas sensor using low UV power (3 μW/cm^2^).
(iii) Detection of NO at a minimum concentration of 4 ppb at room temperature.

## Conclusions

A sensor for NO_*x*_ detection was demonstrated, and a novel nanoporous TiO_2_/SnO_2_ heterojunction was created using a two-step sol–gel process, with reduced power consumption and cost-effectiveness. The UV-activated porous TiO_2_/SnO_2_-based sensor demonstrates an impressive LOD for NO gas at 4 ppb at room temperature while operating at a low voltage of 1 V. This sensor requires minimal UV power (3 μW/cm^2^) to function as a photocatalyst, significantly enhancing gas sensing performance compared to the existing literature. It shows high sensitivity to NO concentrations below 100 ppb, making it ideal for detecting NO in human breath. The sensor’s ability to operate across a wide relative humidity range (10–60% RH) increases its applicability in various environmental conditions. UV illumination improves recovery times by 7-fold and extends the sensor’s lifetime up to 30 days compared to operation without UV. Oxygen vacancies and photogenerated electron–hole pairs play crucial roles in adsorbing oxygen species and enhancing NO_*x*_ attachment on the sensing film. Finally, the ultralow UV power required can be harvested from sunlight, enabling the sensor to function effectively without UV LEDs while ensuring full recovery, improved lifetime, and stability. The nanoporous structure is essential for stabilizing the sensor under low UV power at room temperature. Overall, this low-powered, cost-effective, and reliable NO_*x*_ sensor is suitable for both environmental and clinical applications, contributing to reduced carbon emissions and improved sustainability.

## Supporting Information Available

Additional experimental details and characterization data, including TiO_2_/SnO_2_ thin film preparation, gas sensing system setup, fabrication details of sensors, EDS analysis, XPS data, absorption spectra, band gap calculations, LOD calculation, real-time sensor data under UV illumination, dynamic NO/NO_2_ responses, selectivity tests under varying RH, UV stabilization studies, calibration curves for sensor lifetime, and performance comparisons of UV-activated gas sensors (PDF)
