# Supplementary material for: Highly Efficient UV-Activated TiO2/SnO2 Surface Nano-matrix Gas Sensor: Enhancing Stability for ppb-Level NOx Detection at Room Temperature
Source: ACS Appl Mater Interfaces. 2025 Feb 19;17(9):14670–81. doi: 10.1021/acsami.4c19998 (PMC11891840; doi:10.1021/acsami.4c19998)
Supplement: Supplementary file 1 — am4c19998_si_001.pdf [file am4c19998_si_001.pdf]

Supporting information

High Efficient UV Activated TiO_2/SnO_2 Surface Nano-Matrix Gas Sensor: Enhancing Stability for ppb-Level NOx Detection at Room Temperature

Moumita Deb[1,2,3], Youssef Ghossoub[4,5], Laurent Noel[4,5], Pin-Hsuan Li[2,3], Hsu-Yang Tsai[2,3], Olivier Soppera[4,5*], and Hsiao-Wen Zan[2,3*]

[1] International Ph.D. Program in Photonics, College of Electrical and Computer Engineering, National Yang Ming Chiao Tung University, 1001 Ta Hsueh Rd. Hsinchu 300093, Taiwan.

[2] Department of Photonics, National Yang Ming Chiao Tung University, 1001 Ta Hsueh Rd. Hsinchu 300093, Taiwan.

[3] Department of Photonics, National Chiao Tung University, 1001 Ta Hsueh Rd. Hsinchu 300093, Taiwan.

[4] Université de Haute-Alsace, CNRS, IS2M UMR 7361, F-68100 Mulhouse, France

[5] Université de Strasbourg, F-67000 Strasbourg, France

Corresponding authors email: hsiaowen@nycu.edu.tw; olivier.soppera@uha.fr

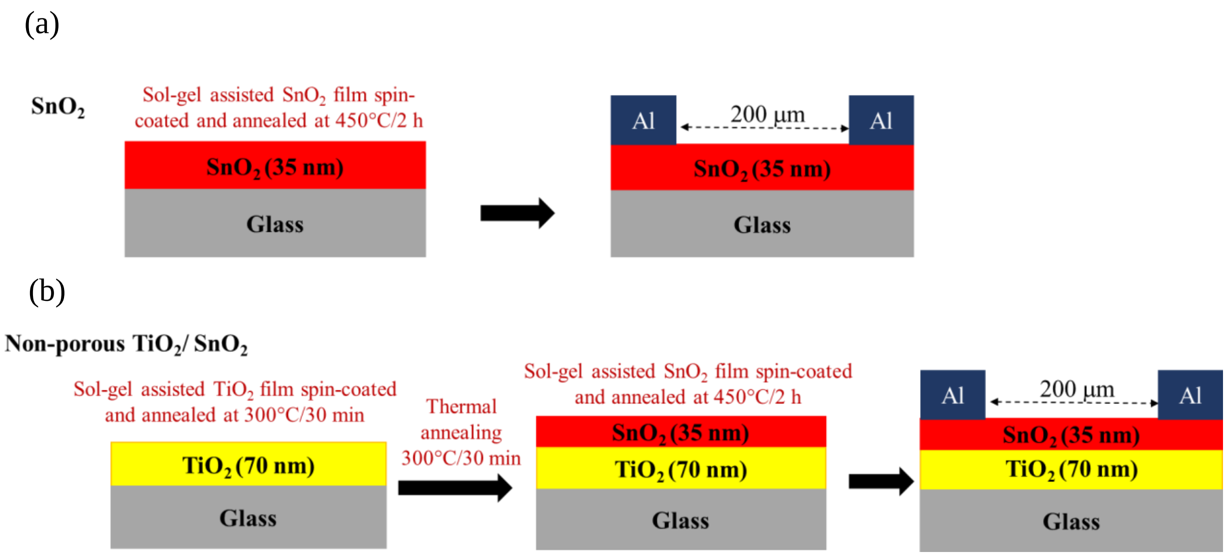

Figure S1: Schematic of fabrication process of (a) SnO_2, and (b) Non-porous $TiiO_2/SnO_2$-based sensor

S1. Gas Sensing Measurement Setup: The gas sensing measurement system was used similarly to our prior works [42, 43]. Relative humidity was controlled using dry and wet air, and a UV LED light (365 nm) was added on top of the glass chamber (Figure S2). Power was supplied to the LED through a Keithley 2400 with currents of 1, 1.5, 2, 3, and 4 mA to achieve power intensities of 3, 10, 20, 30, and 40 $\mu W/cm^2$, respectively. For UV-activated gas sensing measurements (Figure S2), a variety of instruments were utilized. An air cylinder connected to a mass flow controller was used to regulate the relative humidity (RH) in the sensing chamber, which was maintained at adjustable levels ranging from 10% to 60%. The current-voltage (I-V) characteristics of the fabricated sensors were measured at room temperature (24±1 °C) using a source meter unit (Tektronix Keithley 2400). A single-channel syringe pump (LSP01-1A) was employed to introduce the target gases, NO and NO_2, at concentrations of 100 ppm and 200 ppm, respectively. The gas in the syringe was mixed with background air at a flow rate of 500 mL/min and adjustable RH levels.The total outlet flow rate was maintained at 500 mL/min, which was monitored using a flow meter. This air-gas mixture, with controlled RH, was directed into a glass chamber where the sensor was exposed to the NOx gas along with the background air mixture. The concentration of the gas in the chamber, ranging from 4 ppb to 1000 ppb, was controlled by adjusting the volume and flow rate of the syringe pump. A flow meter (Dwyer, 1000 cc/min) and a micro pump were used to maintain constant pressure in the chamber, while the RH level was monitored using a thermo-hygrometer (CHY 321). A UV LED (365 nm) with a fixed power intensity of 3 $\mu W/cm^2$ was positioned at the top of the sensing chamber to activate the photocatalytic response.

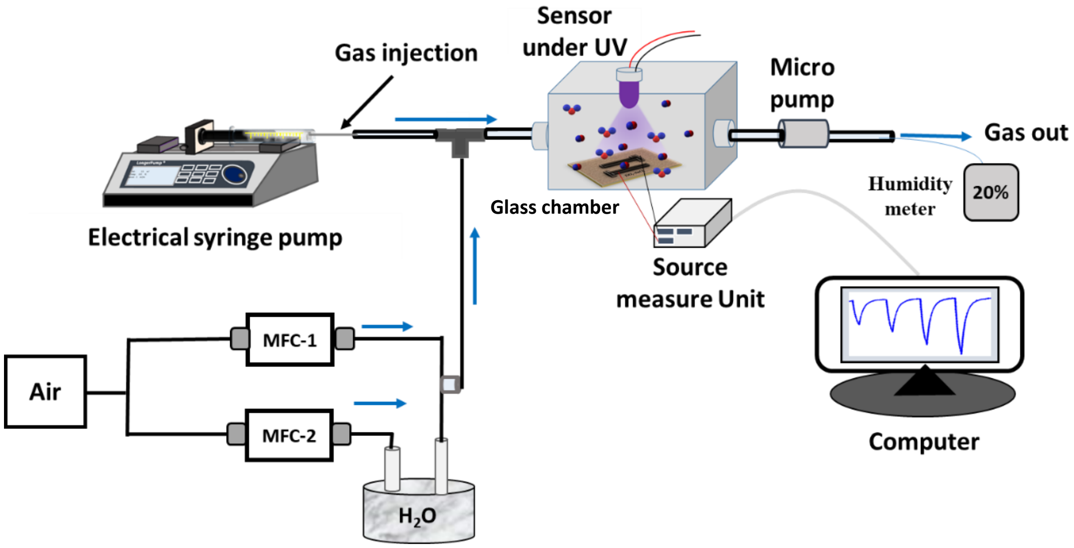

Figure S2: UV-activated gas sensing measurement system

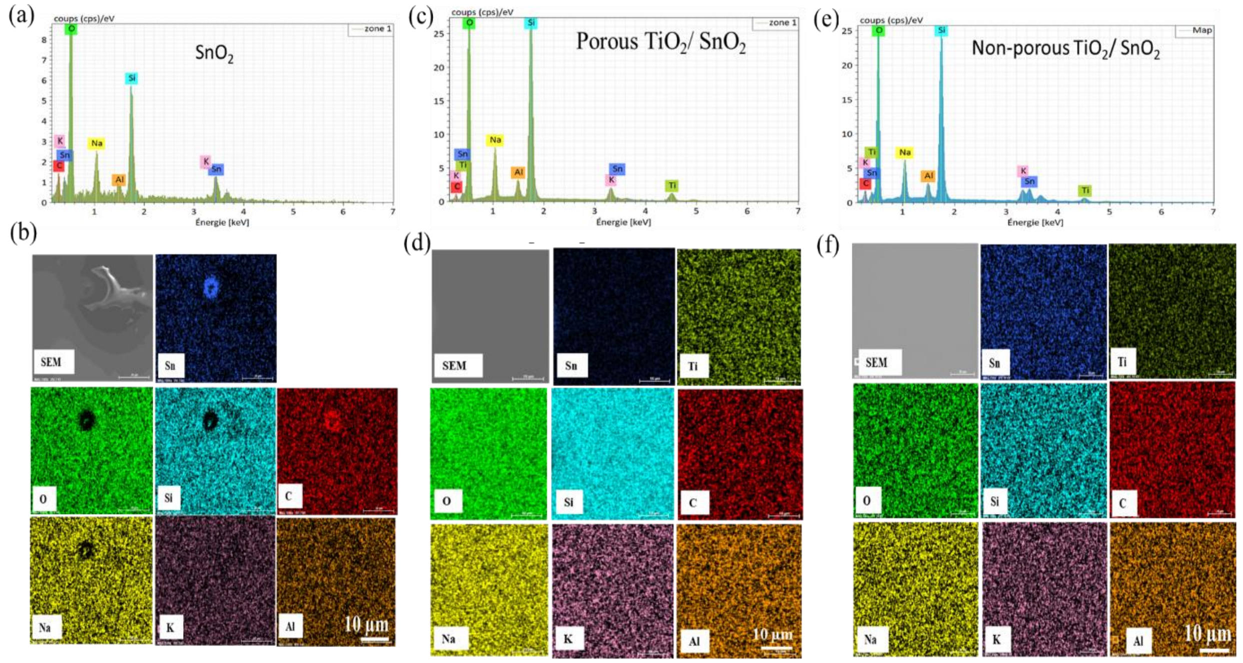

Figure S3 Elemental analysis: (a, c, e) EDX spectrum of SnO_2, Porous TiO_2/SnO_2, and Non-porous TiO_2/SnO_2 films; (b, d, f) EDX elemental mapping of SnO_2, Porous TiO_2/SnO_2, and Non-porous TiO_2/SnO_2 films.

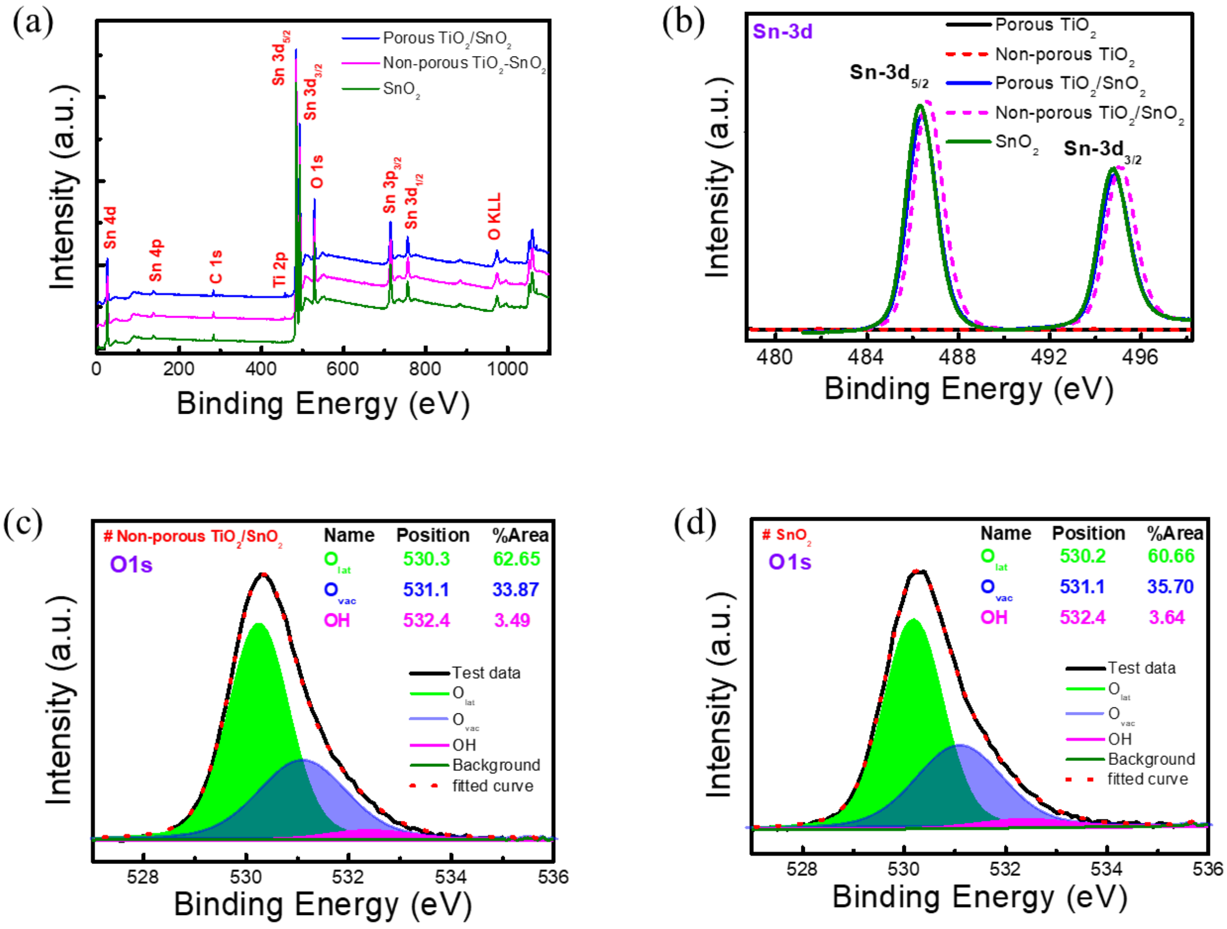

Figure S4: XPS images of (a) SnO_2 and TiO_2/SnO_2 film all chemical analysis, (b) Sn-3d component analysis. (c) O1s component of SnO_2 and (d) Non-porous TiO_2/SnO_2 film.

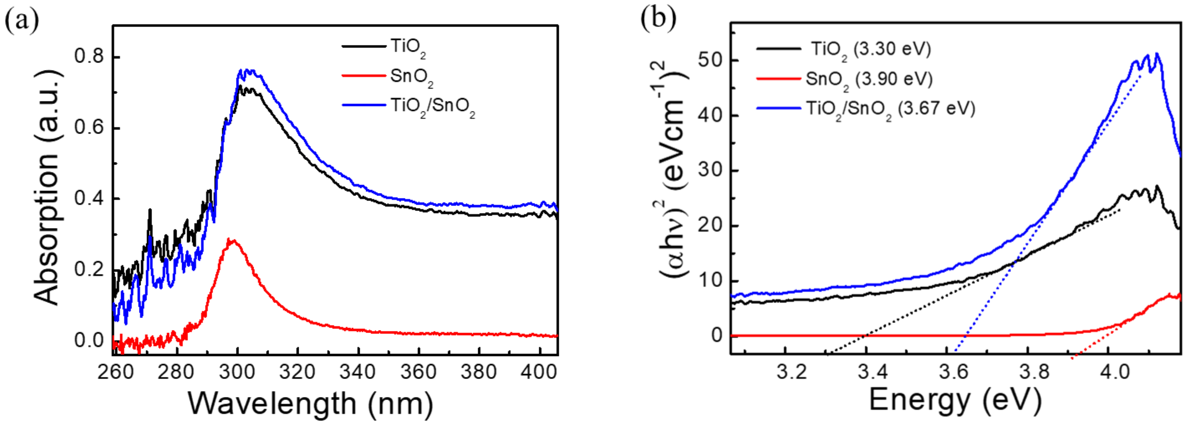

Figure S5: (a) Absorption spectra and (b) calculation of energy gap of TiO_2, SnO_2 and TiO_2/SnO_2 film from absorption data.

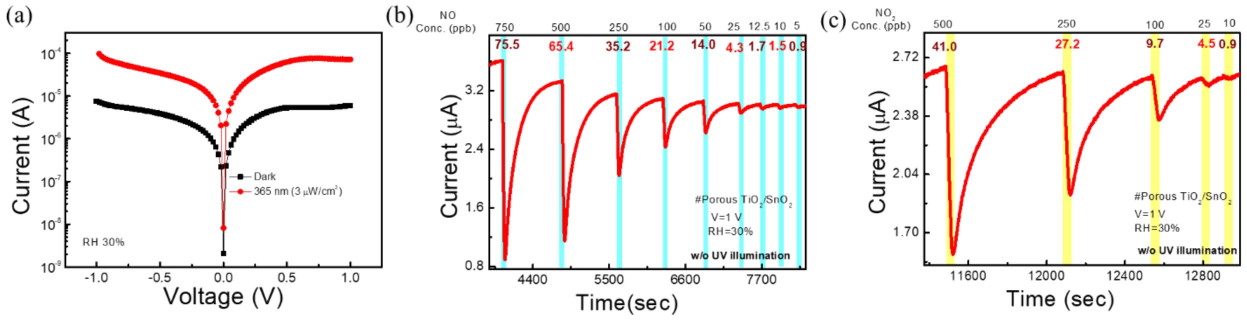

Figure S6: (a) I-V curve of porous TiO_2/SnO_2 based sensor. (b) Dynamic response data of NO and (c) NO_2 w/o UV illumination

S2: Calculation of LOD: The limit of detection (LOD) was determined using the calibration curve, which was based on the sensor's response to different concentrations of NO and NO_2 gases (10, 25, and 50 ppb). The calibration curve was linearly fitted using Origin software, providing key parameters essential for calculating the LOD of NO and NO_2 gases. These important parameters are listed below, followed by the detailed LOD calculation.

**Different parameters**	**NO**	**NO_2**
Residual sum of squares (from origin data)	0.00255	0.09184
Slope (from origin data)	0.23776	0.17347
Ads. R^2 value (from origin data)	0.99989	0.99255
LOD = 3* sqrt (residual sum of squares)/slope (ppb)	0.637	2.4

LOD = 3* standard deviation/slope S(1)

= 3* sqrt (residual sum of squares)/slope

= 3* sqrt (0.00255)/0.23776

= 0.637 ppb

= 637 ppt

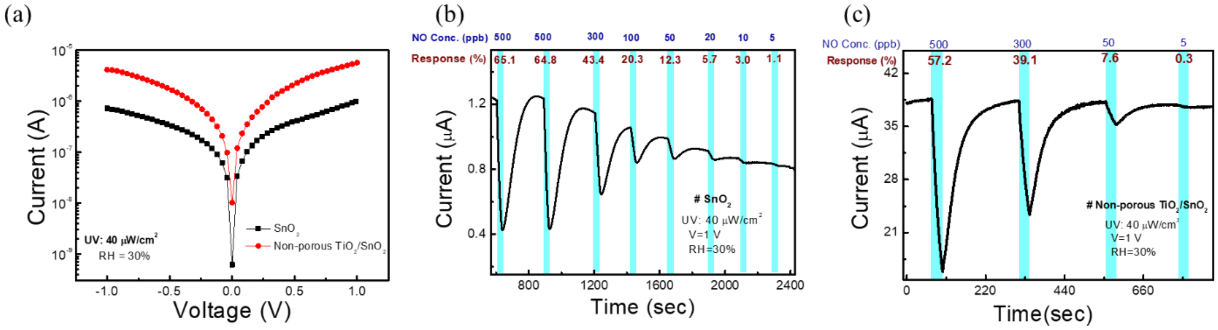

Figure S7: (a) I-V curve of SnO_2 and Non-porous TiO_2/SnO_2 based sensor under UV illumination (40 W/cm^2) (b) SnO_2-based and (c) Non-porous TiO_2/SnO_2 based dynamic NO gas response data from 5 to 500 ppb under UV.

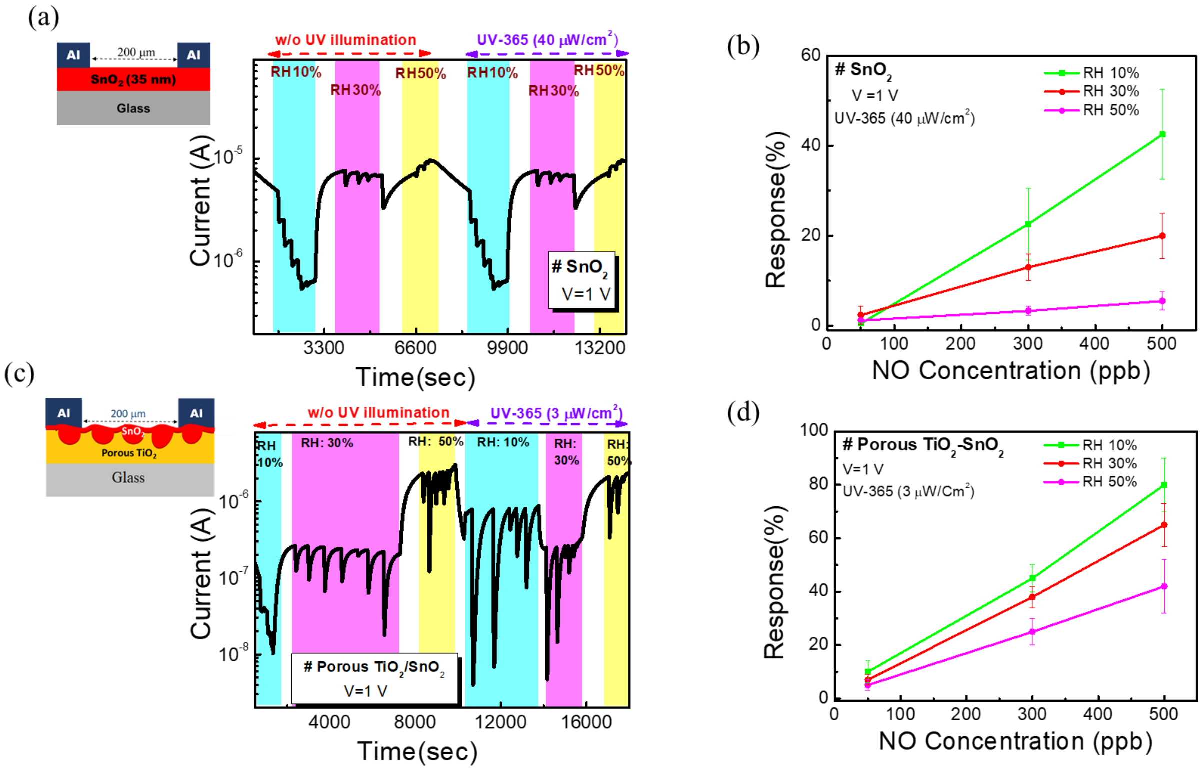

Figure S8: Dynamic NO sensing data for (a) SnO_2, and (c) porous TiO_2/SnO_2-based sensors without UV and under UV illumination at different RH levels. The response vs. NO concentration calibration curve for different sensors: (b) SnO_2 (UV: 40 µW/cm²), and (d) porous TiO_2/SnO_2 (UV: 3 µW/cm²), at different RH levels.

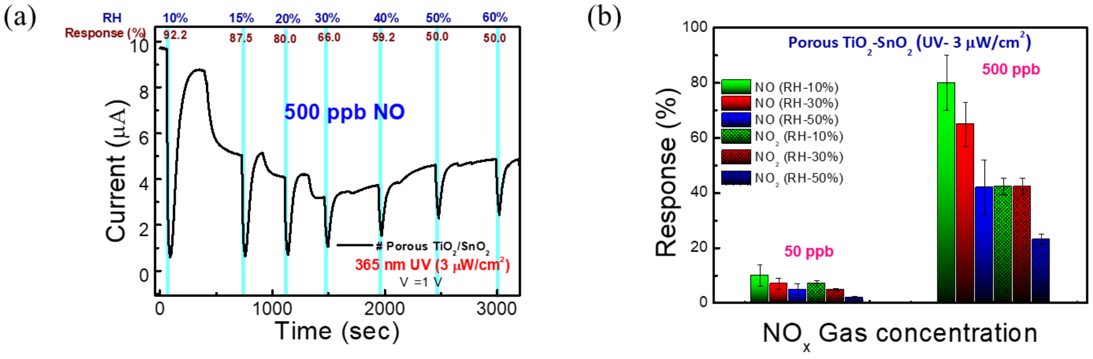

Figure S9: (a) Real-time dynamic NO response data of the porous TiO_2/SnO_2-based sensor under varying RH (10 to 60%) at 500 ppb gas concentration. (b) RH effect on NOx (NO and NO_2) gas response of the porous TiO_2/SnO_2-based sensor at 50 and 500 ppb gas concentrations under UV illumination.

S3. Recovery ratio: The recovery ratio (r) is defined as the proportion of the restored signal to the original signal following exposure to the gas. This ratio represents the change in current when exposed to the gas compared to the change in current in air. It serves as an indicator of the sensor's capability to revert to its baseline state after encountering the target gas. A higher recovery ratio suggests quicker recovery and enhanced sensor stability.

$$r(\%) = \frac{I_g - I_r}{I_g - I_a} \times 100\% \qquad \text{S(2)}$$

Where I_r is the current at the end of the sensing cycle, and I_g and I_a are defined as the current under gas exposure and in background air, respectively.

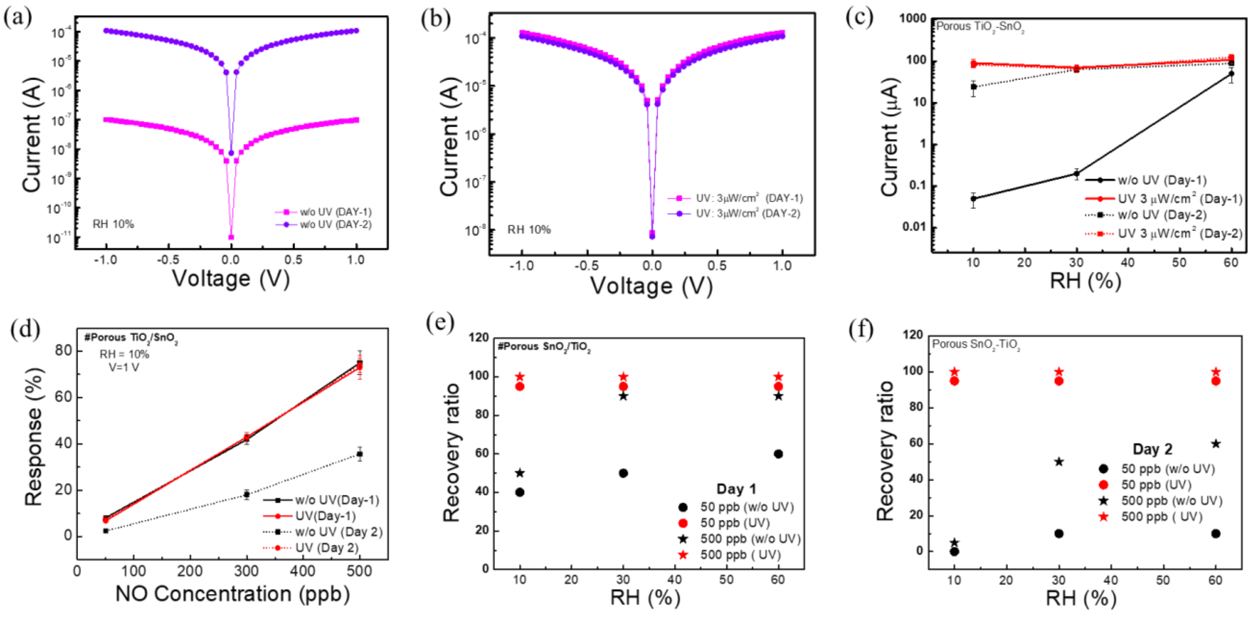

Figure S10: Importance of UV illumination for stabilizing background current: (a) I-V curve of the porous TiO_2/SnO_2-based sensor without UV illumination and (b) under UV illumination (3 μW/cm^2) on day 1 and day 2 at RH 10%. (c) Background current vs. RH curve. (d) Importance of UV illumination for stabilizing response, shown by NO concentration vs. response calibration curve on day 1 and day 2 at RH 10%. (e) Importance of UV illumination for improving recovery ratio, illustrated by sensor recovery under different humidity levels on (f) day 1 and day 2.

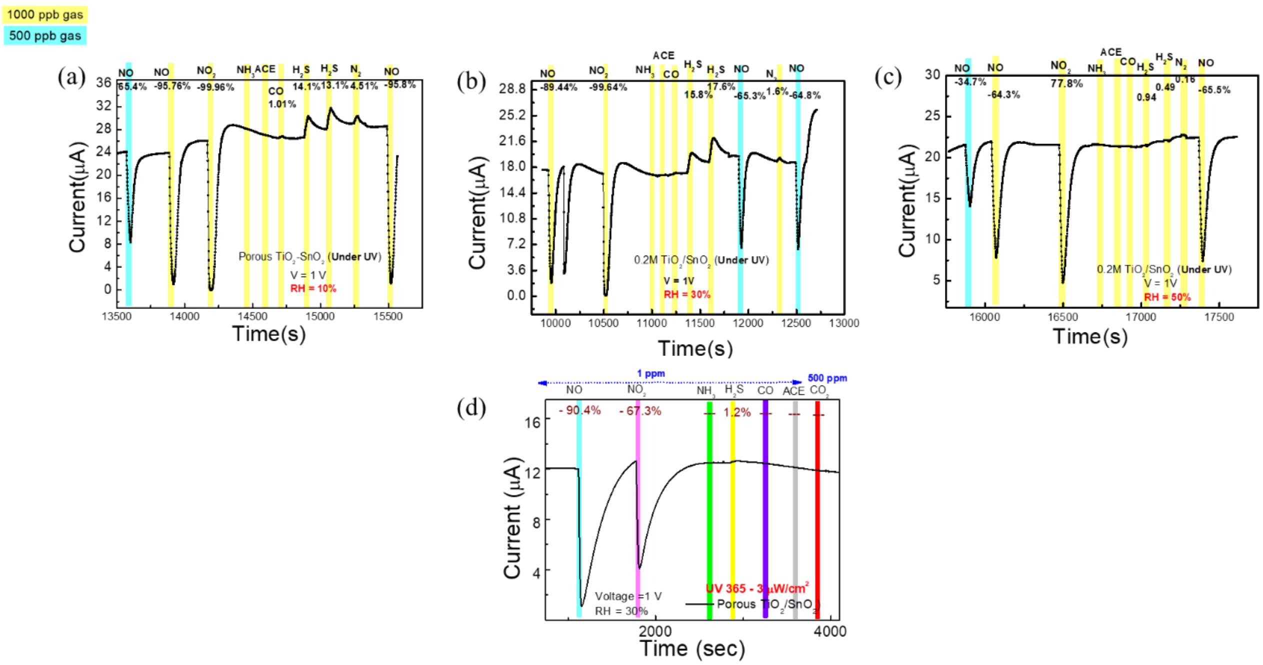

Figure S11: Real time gas response data of different gasses to check the selectivity of porous TiO_2/SnO_2 based sensor at different humid medium (a) 10% RH (b) 30% RH and (c) 50% RH. (d) Selectivity test of 1 ppm NOx, H_2S, CO, Acetone, NH_3 and 500 ppm CO_2 gases under 30% RH.

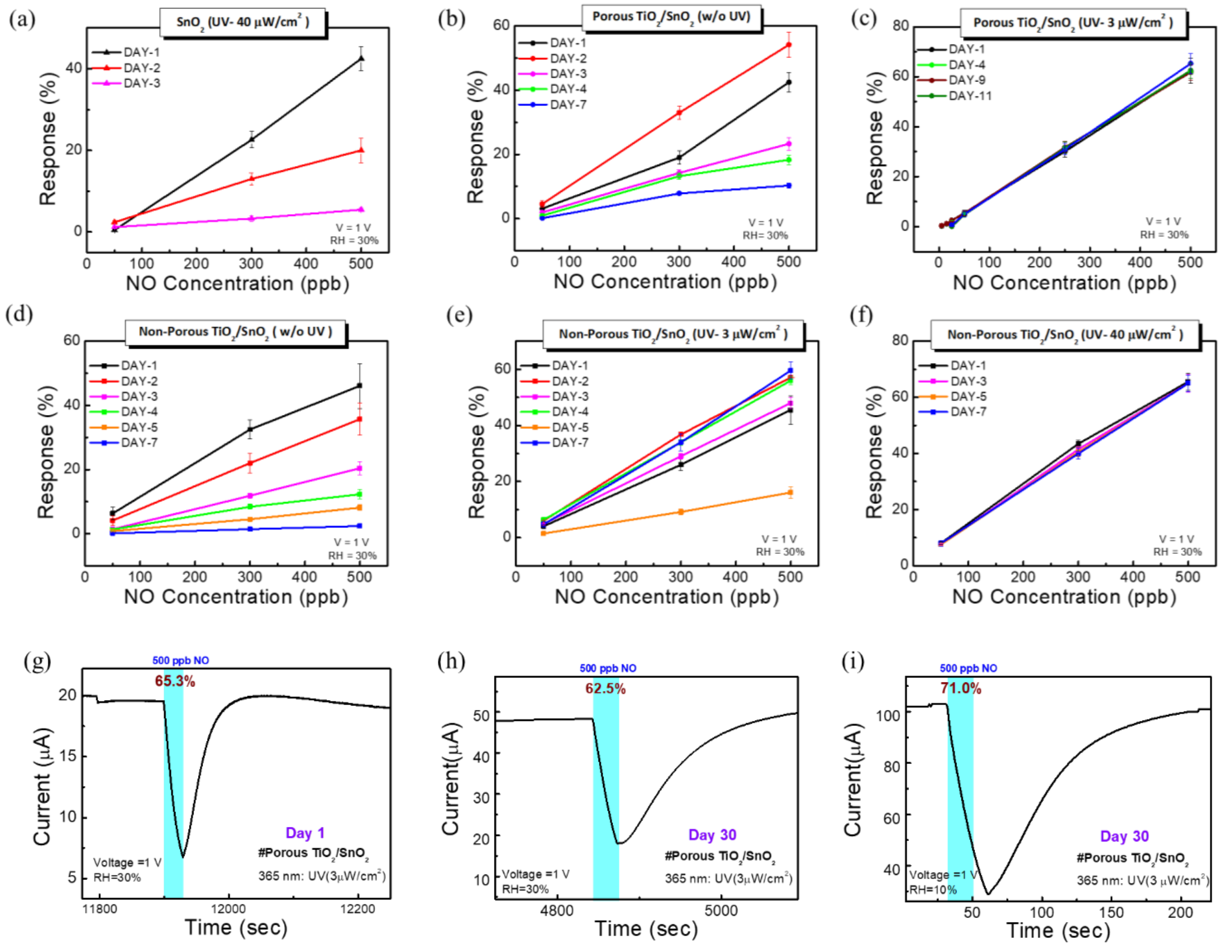

Figure S12. Life time data (RH 30%) of (a) SnO_2 und UV 40 μW/cm^2 , (b) Porous TiO_2/SnO_2 w/o UV, and (c) under UV 3 μW/cm^2. (d) Non-porous TiO_2/SnO_2 w/o UV, (e) under UV 3 μW/cm^2 and (f) under UV 40 μW/cm^2. (g) NO gas response real-time data for a 500 ppb concentration using the porous TiO_2/SnO_2 sensor measured on Day 1 and (h) Day 30 under UV 3 μW/cm^2. (i) Real-time lifetime data at 10% RH under UV illumination on day 30.

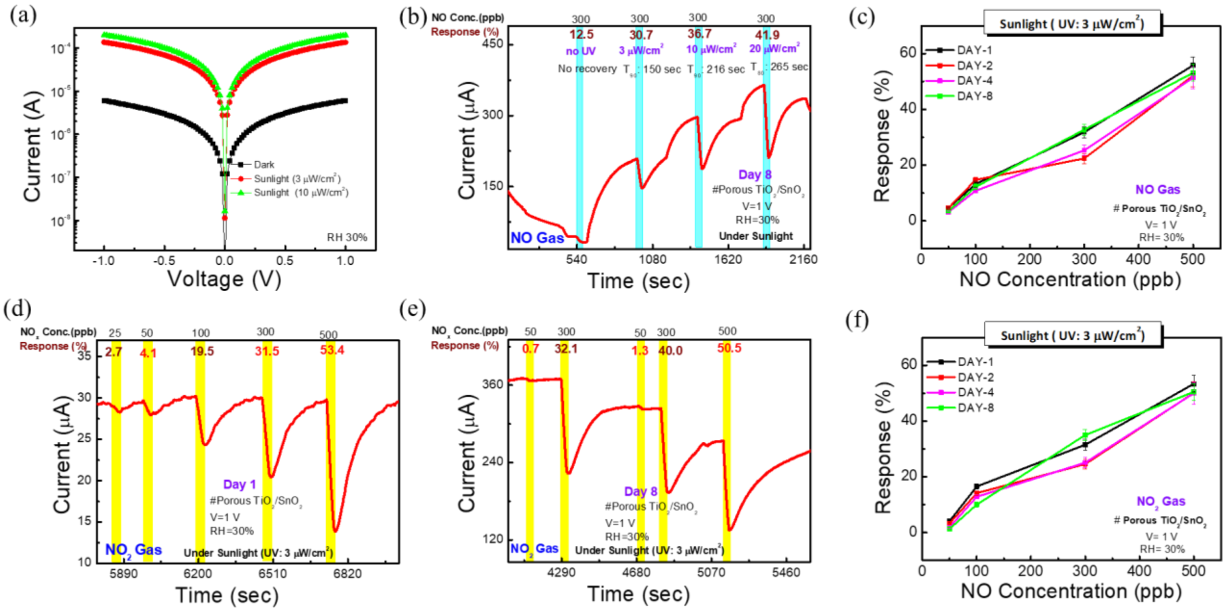

Figure S13: Performance of the porous TiO_2/SnO_2-based NOx sensor under varying sunlight intensities. (a) I-V characteristics measured at different sunlight intensities. (b) Real-time dynamic response of the sensor to 300 ppb NO gas under various sunlight power intensities. (c, f) Calibration curves for NO and NO_2 gases, with concentrations ranging from 50 to 500 ppb, monitored over 8 days at a constant sunlight intensity of 3 μW/cm². (d, e) Real-time response data for NO_2 gas recorded on day 1 and day 8.

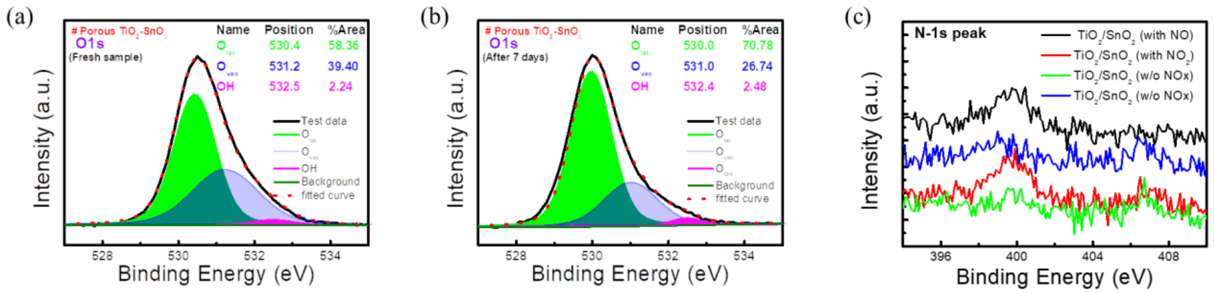

Figure S14. XPS data showing the decrease in oxygen vacancies (O_{vac}) over time: (a) O1s spectrum of porous TiO_2/SnO_2 on day 1 (fresh sample) with O_{vac} at 39.40%, and (b) on day 7 with a decreased O_{vac} at 26.74%. (c) Appearance of the N1s peak before and after NOx gas injection on the porous TiO_2/SnO_2 film.

Table S1: Comparison of UV activated different gas sensors at room temperature.

Sensing Materials	Sensing Method	Working Temp.	UV wavelength (Power)	Gas	Gas Con. (ppm)	Response	LOD	Year Ref.
TiO_2@SnO_2	Hydrothermal	RT	365 nm (2.5 mW/cm^2)	HCHO	10	20	100 ppb	2020 [18]
TiO_2@NGQDs	Hydrothermal	27 °C	365 nm (3.2 mW/cm^2)	NO	100	30%	10 ppm	2020 [20]
AuNPs-decorated ZnO/TiO_2 NRs	Spin coating Sol-gel	RT	370 nm (1.2 mW/cm^2)	NO_2	50	7.5	10 ppm	2021 [21]
TiO_2 NPs/PrGO	Drop casting	27 °C	365 nm (5.34 mW/cm^2)	NO_2	100	35.6	114 ppb	2021 [22]
CuO/TiO_2	Thin film	RT	365 nm (17−48 mW/cm^2)	H_2S	100	46.81%	3 ppm	2021 [23]
SnO_2	ALD deposition	RT	275 nm (150 µW/cm^2)	CO	50	56.7	100 ppb	2022 [19]
SnO_2/TiO_2	ALD deposition	RT	275 nm (50 µW/cm^2)	HCHO	10	32.7	1 ppm	2022 [19]
Nano-porous TiO_2/SnO_2	Sol-gel	RT	365 nm (3 µW/cm^2)	NO	1	~96%	4 ppb	This work
Thin film TiO_2/SnO_2	Sol-gel	RT	365 nm (40 µW/cm^2)	NO	1	~90%	5 ppb	This work
Nano-porous TiO_2/SnO_2	Sol-gel	RT	365 nm (3 µW/cm^2)	NO_2	1	~87%	10 ppb	This work

Table S2: Comparison table of NO gas sensor for ppb level LOD detection.

Sensing Materials	Fabrication method	Sensor preparation temp.	Elec-trode	Working Temp. (◦C)	Gas conc. (ppm)	Res-ponse	Tres/ Trec (sec)	LOD (ppb)	Refs.
ZnO nanobelts	Carbo thermal reduction method	1050 °C/ 5 h 500 °C/ 1 h	Au	28	10	1.7	135/ 130	500	[28]
$Nb_2O_5/In_2O_3/WO_3$	PLD	900 °C/ 6 h	Au	70	0.1	56.1	1911/ 593	20	[29]
ZnO NRs	Sputtering	--	Ag NWs /ITO	RT	1	57.5 %	30/-	100	[30]
Bi-SnO_2 nanosheets	Electro-spinning	600 °C/ 2 h	Au	75	5	90	100/ 120	50	[31]
Pd@Fe_2O_3/ MWCNTs/ WO_3	So-gel	500 °C/ 4 h	Au	25	0.5	1.18	291/ 511	100	[32]
ZnO microtubules	Bio-derived sol-gel	500, 600 and 700 ◦C for 2 h	Au	92	10	78.54	56/ 92	5	[33]
P-type TiO_2 film (TSV)	ALD/RF sputtering	---	Al	RT	4	16.7 %	109/ 144	500	[34]
GC/SnO_2 tubes	Bio-derived sol-gel	700 ℃/2 h	Au	50	1	256.3	100/ 42	50	[35]
Nano-porous TiO_2/SnO_2	Sol-gel	450 ℃/2 h	Al	RT :UV	1	96%	30/95	4	This work
Thin film TiO_2/SnO_2	Sol-gel	450 ℃/2 h	Al	RT : UV	1	95%	30/96	5	This work

Table S3: Dynamic gas response of TiO_2 /SnO_2-based sensor under 50 ppb NO gas at different injection times (30 to 240 seconds) with response/recovery time to assess long-term detectability.

Injection time (sec)	Max. Response (%)	Response time (τ_{90}) (sec)	Recovery time (τ_{90}) (sec)
**30**	7.5	35	85
**60**	13.4	54	100
**90**	18.1	85	190
**120**	21.9	110	320
**150**	25.9	145	310
**180**	28.8	171	375
**210**	31.1	201	420
**240**	33.9	233	410
